# Probing molecule-like isolated octahedra via phase stabilization of zero-dimensional cesium lead halide nanocrystals

**DOI:** 10.1038/s41467-018-07097-x

**Published:** 2018-11-08

**Authors:** Paulraj Arunkumar, Han Bin Cho, Kyeong Hun Gil, Sanjith Unithrattil, Yoon Hwa Kim, Won Bin Im

**Affiliations:** School of Materials Science and Engineering, Chonnam National University, 77, Yongbong-ro, Buk-Gu, Gwangju 61186 Republic of Korea

## Abstract

Zero-dimensional (0D) inorganic perovskites have recently emerged as an interesting class of material owing to their intrinsic Pb^2+^ emission, polaron formation, and large exciton binding energy. They have a unique quantum-confined structure, originating from the complete isolation of octahedra exhibiting single-molecule behavior. Herein, we probe the optical behavior of single-molecule-like isolated octahedra in 0D Cesium lead halide (Cs_4_PbX_6_, X = Cl, Br/Cl, Br) nanocrystals through isovalent manganese doping at lead sites. The incorporation of manganese induced phase stabilization of 0D Cs_4_PbX_6_ over CsPbX_3_ by lowering the symmetry of PbX_6_ via enhanced octahedral distortion. This approach enables the synthesis of CsPbX_3_ free Cs_4_PbX_6_ nanocrystals. A high photoluminescence quantum yield for manganese emission was obtained in colloidal (29%) and solid (21%, powder) forms. These performances can be attributed to structure-induced confinement effects, which enhance the energy transfer from localized host exciton states to Mn^2+^ dopant within the isolated octahedra.

## Abstract (web summary)

Zero-dimensional cesium lead halides show interesting optoelectronic properties owing to the isolated octahedral structure, but their interaction with alien atoms remain elusive. Herein, Arunkumar et al. report manganese ion doping which leads to phase stabilization and higher quantum yield.

## Introduction

Over the past few years, inorganic lead halide perovskites have attracted great attention as a promising optoelectronic material for light-emitting devices, photodetectors, and low-threshold lasers, owing to their high photoluminescence quantum yield (PL QY), narrow emission, and tunable band gap^1–4^. The basic building block in these material is PbX_6_ octahedra (where X is a halogen), whose diverse connectivity can produce structures with various dimensionalities, ranging from three-dimensional (3D) to zero-dimensional (0D)^5–7^. The 3D inorganic perovskites, with general formula APbX_3_ (A = Cs, Rb, and X = Cl, Br, or I), consist of an extended network of corner-sharing PbX_6_ octahedra with cavities occupied by A ions^8,9^. Despite being the most explored material, the poor chemical stability of 3D perovskites against moisture, inherent phase transformation, and ion migration make their low-dimensional counterparts more favorable for optoelectronic applications^4,6,10^.

In particular, 0D inorganic perovskite-like Cs_4_PbX_6_ system have crystal structure in which the PbX_6_ octahedra are decoupled from each other by the surrounding Cs^+^ ions. The complete isolation of octahedra leads to strong quantum confinement and exciton–phonon interactions, which in turn can result in exciton localization, self-trapping, and polaron formation^10^. The optical features of 0D Cs_4_PbX_6_ are governed by transitions between the electronic states of Pb^2+^ ions, and its broad ultraviolet (UV) emission has been assigned to the radiative decay of Frenkel excitons at Pb^2+^ sites^11–15^. However, the origin of their PL in the visible range is still under debate because of mixed views on the efficient green luminescence (QY of 45%) of Cs_4_PbBr_6_, which some studies have been attributed to the minor 3D CsPbBr_3_ nanoscale impurity^3,16–18^.

Nikl et al.^12^ reported a UV emission band at 355 nm for Cs_4_PbCl_6_ single crystals, assigning to Pb^2+^ ion emission, originating from the optical transitions of ^3^P_0_,_1_ → ^1^S_0_ in the isolated PbCl_6_ octahedra, similar to Pb^2+^ doping in the alkali halide hosts^12^. In addition, the coexistence of 3D CsPbCl_3_-like impurity was noted by 414 nm emission in Cs_4_PbCl_6_. While, Mohammed’s group^19^ reported Cs_4_PbBr_6_ characteristic with two broad UV emissions at 340 nm (high-energy) and 400 nm (low-energy) attributing to Pb^2+^ ion and charge-transfer band denoted as “D-state”, respectively. D-state emission originates via the transfer of excited electrons from (PbBr_6_)^4^^–^ octahedra to the Pb^2+^ occupying Cs sites (D-states), through their strong coupling in Cs_4_PbX_6_ host^19^. Therefore, 0D Cs_4_PbX_6_ behave as an ideal host–guest system, with periodically doped individual PbX_6_ species in a wide band gap matrix. However, though many reports attribute the green emission (512 nm) to the intrinsic property of Cs_4_PbBr_6,_ we believe it arise from the coexistence of CsPbBr_3_ impurity, similar to 414 nm emission of Cs_4_PbCl_6_^12^. Nevertheless, 0D cesium lead halide perovskites still represent largely unexplored and intriguing perovskite-like material, expected to exhibit interesting optoelectronic properties, due to their strongly localized excitons and high exciton binding energies (150 to 380 meV)^3,20,21^.

The coexistence of 3D CsPbX_3_ impurity has precluded a detailed understanding on the optical properties of Cs_4_PbX_6_, leading to a growing demand for their synthesis in pure form and their phase stabilization. Solvent washing has been recently reported for the synthesis of Cs_4_PbBr_6_ free from CsPbX_3_ impurity, which, however, is inadequate to treat the chloride compositions due to their poor solubility^3^. As the inevitable coexistence of CsPbX_3_ phase represents a major drawback, new synthetic strategies are of utmost interest for obtaining pure Cs_4_PbX_6_ nanocrystals with reduced CsPbX_3_ impurity.

Recently, the molecule-like behavior of Cs_4_PbBr_6_ has been discussed in terms of charge carrier transport and polaron formation, arising from weak interactions between isolated octahedra^10^. Thus, understanding the fundamental optoelectronic behavior of 0D Cs_4_PbX_6_ would promote the development of bulk perovskite-based devices. Incorporation of transition metal ions such as manganese (Mn^2+^) in the semiconductor nanocrystal leads to interesting optoelectronic properties by modulating the electronic properties of the host, providing new routes for designing solid-state lighting and light-harvesting devices^22,23^. Hence, Mn^2+^ ions can serve as the sensitive probe for investigating the local structure of the host and altering its optical and electronic behavior. Thus far, the potential effect of isovalent cation doping on the isolated octahedral units of Cs_4_PbX_6_ remains unexplored, particularly in terms of phase stabilization and optical properties.

In this work, we probe the optical behavior of isolated octahedra in the Cs_4_PbX_6_ (X = Br, Cl, Br/Cl) by introducing Mn^2+^ at the octahedral sites and explore its phase stabilization over commonly coexisting CsPbX_3_ impurity. The luminescence measurements show that Mn^2+^ dopant significantly alter the optical properties of different host emissive states in the Cs_4_PbX_6_. The dopant emission is controlled by adjusting the band gap of Cs_4_PbX_6_ through halide identity, facilitating energy transfer for enhancing the dopant emission efficiency in both colloidal and solid forms. The quantum-confined structure of 0D Cs_4_PbX_6_ further improves the stability of the host and efficiency of the dopant emission, particularly in their solid form.

## Results

### Mn^2+^-doped Cs_4_PbX_6_ nanocrystals

A series of 0D Cs_4_PbX_6_ (X = Br, Br/Cl, and Cl) nanocrystals, doped with manganese, were synthesized via modified reverse microemulsion method (Supplementary Figure 1 and Supplementary Note 1)^20^. A short-chain alkylamine (octylamine) surfactant was employed instead of the previously reported long-chain oleylamine to control size and morphology^20^. The exact composition of the synthesized undoped and Mn^2+^-doped Cs_4_Pb(Br/Cl)_6_ (denoted as mixed halide) analogs are Cs_4_PbBr_2_Cl_4_ and Cs_4_Pb_1__–__*x*_Mn_*x*_Br_2–2*x*_Cl_4+2*x*_, respectively, with *x* varying from 0.05 (5% Mn) to 0.80 (80% Mn). The Pb/Mn ratios were estimated from inductively coupled plasma−optical emission spectroscopy (Supplementary Table 1). For simplicity, the synthesized samples were represented as Cs_4_PbX_6_:*x*% Mn, where X = (Br/Cl), Cl, and Br; and *x* = percentage of Mn concentration with respect to displaced lead ions.

### Local and bulk structure

**Fig. 1:**
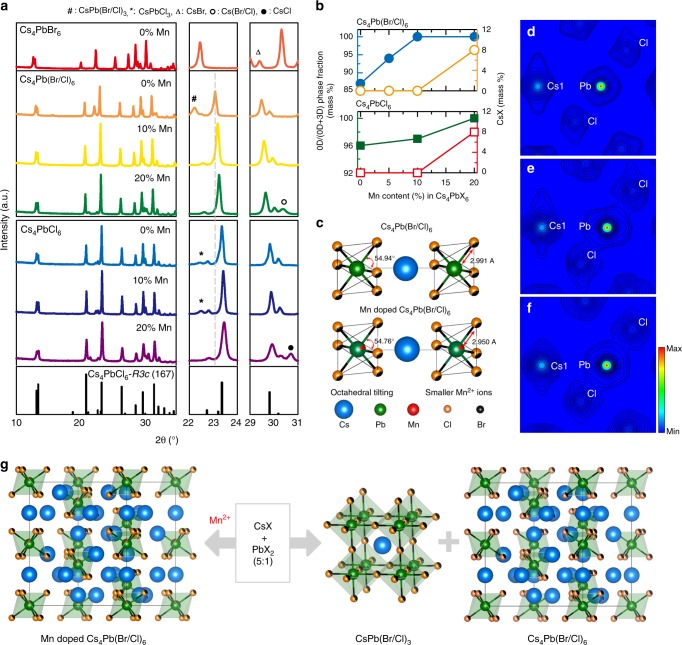
Local and bulk structures of Mn^2+^-doped Cs_4_PbX_6_. **a** X-ray diffraction patterns of Mn^2+^-doped Cs_4_PbX_6_ series with different doping concentrations showing the rhombohedral structure is preserved after Mn-doping. Selected 2*θ* range of (22 to 24° and 29 to 31°) XRD patterns were presented. A monotonic shift of the XRD peak at Bragg’s angle at 23.4° towards higher 2*θ* is the result of progressive lattice contraction as the Mn^2+^ concentration increases, due to the substitutional replacement of Pb^2+^ with isovalent but smaller Mn^2+^ ions. The additional impurity cubic phases of 3D CsPb(Br/Cl)_3_, 3D CsPbCl_3_, CsBr, Cs(Br/Cl), and CsCl were denoted as hash, asterisk, triangle, open circle, and closed circle, respectively. It is to be noted that the Cs:Pb precursor ratio was fixed at 5:1 for all the samples of Cs_4_PbX_6_ (Br, Cl, and Br/Cl). **b** Phase fraction of the desired 0D Cs_4_PbX_6_ perovskite with respect to 3D CsPbX_3_ perovskite phase and segregation of CsX, with varying Mn concentration in the Cs_4_PbX_6_ synthesis. **c** Octahedral tilting/distortion without and with Mn doping (10% Mn) causing the phase stabilization of Cs_4_Pb(Br/Cl)_6_ structure. Electron density distribution profile derived from Maximum Entropy method for the **d** undoped Cs_4_PbCl_6_, **e** Cs_4_PbCl_6_:10% Mn, and **f** Cs_4_PbCl_6_:20% Mn, along the (010) plane. **g** Schematic representation of phase stabilization of Cs_4_PbX_6_ perovskite structure upon Mn^2+^ incorporation

The crystal structure of Cs_4_PbX_6_ consists of caged PbX_6_ octahedra, isolated by interspersed Cs–X bridges^21^. Two types of Cs sites are present: the Cs(1) site form an alternating octahedra with PbX_6_, whereas the Cs(2) trigonal prisms share one triangular face to form infinite [CsPbX_6_]_n_^3^^–^ chains along the [001] direction^24^. The synthesized samples were highly crystalline matching with the rhombohedral Cs_4_PbX_6_ phase (X = Br, Br/Cl, and Cl; space group: *R*$\bar 3$*c*) as presented in Fig. 1a. However, in the undoped Cs_4_PbX_6_, the presence of 3D CsPbX_3_ impurity were clearly apparent in all the halide analogs (Fig. 1a, Supplementary Figure 2, and Supplementary Note 2). Despite using high Cesium content (Cs:Pb = 5:1), formation of undesired CsPbX_3_ phase were observed in the undoped Cs_4_PbX_6_ probably due to localized occurrence of Cs-deficient regions.

Upon Mn incorporation, the Bragg’s peak at 23° monotonically shifts to higher 2*θ*, suggesting the substitution of Pb^2+^ with smaller Mn^2+^ ion in Cs_4_PbX_6_ (X = Br, Br/Cl, Cl), which is also evidenced from the decrease in lattice parameters (Table 1, Supplementary Tables 2 to 4)^24,25^. Lattice contraction of 0.3% and volume change of 2%, after Mn (20% Mn) doping, resulted in the shortening of lead halide (Pb–Br/Cl) bond lengths and similar feature was also noted in chloride analog (Supplementary Table 5, 6). The strong bonding nature upon Mn doping is evidenced from high bond-dissociation energy of Mn–Cl (361 kJ mol^−1^) than Pb–Br bonds (247 kJ mol^−1^)^26^. The valence states and substitution of Mn^2+^ ions at the isolated octahedral PbX_6_ sites was reaffirmed from X-ray photoelectron spectroscopy and electron spin resonance results, respectively (Supplementary Figures 3a to 3f and Supplementary Note 3)^22,27,28^. Quantification of multiple crystalline phases (Cs_4_PbX_6_ and CsPbX_3_) in the synthesized samples were estimated using Rietveld refinement of X-ray diffraction (XRD) results. Interestingly, Mn doping resulted in a decrease in the phase fraction (mass%) of 3D CsPbX_3_ impurity, which further reduced with Mn content in both Br/Cl and Cl analogs, as shown in Fig. 1b. In other words, the phase fraction of 0D Cs_4_Pb(Br/Cl)_6_ with respect to the total (0D+3D) phase gradually increased from 86 to 100% upon increasing Mn concentration from 0 to 20%. This increase in the phase fraction of Cs_4_PbX_6_ was also apparent in the pure chloride and bromide analog (Supplementary Figure 2), suggesting that Mn^2+^ incorporation in the framework favors the formation of Cs_4_PbX_6_ phase, while preventing the undesirable CsPbX_3_ phase. A CsPbX_3_ impurity-free Cs_4_Pb(Br/Cl)_6_ phase was obtained with 10% Mn content. Despite the complete suppression of CsPbX_3_, additional CsX (8%) phase tends to precipitate at higher Mn (above 10% Mn) content in both Cs_4_Pb(Br/Cl)_6_ and Cs_4_PbCl_6_ (Fig. 1b). Since the presence of Mn^2+^ in the lattice favors the Cs_4_PbX_6_ formation, the excess Cs precursor (5:1) which is used to favor the 0D phase, tend to segregate as CsX at higher Mn concentration. The CsX formation could be further prevented by decreasing the Cs precursor (Cs:Pb = 4.5:1) in the Mn^2+^-substituted samples, leading to pure Cs_4_PbX_6_ nanocrystals (Supplementary Figure 4). Our attempt to incorporate higher Mn content above 20% Mn, resulted in an additional hexagonal CsMnCl_3_ phase, along with Cs_4_Pb(Br/Cl)_6_ (Supplementary Figure 5 and Supplementary Note 4).

**Table 1:** Structural parameters of Cs_4_Pb(Br/Cl)_6_ with varying concentration of Mn, calculated using the Rietveld refinement

Formula	Cs_4_Pb(Br/Cl)_6_	5% Mn	10% Mn	20% Mn
Space group	*R* $\bar 3$ *c*	*R* $\bar 3$ *c*	*R* $\bar 3$ *c*	*R* $\bar 3$ *c*
Composition	Cs_4_PbBr_2_Cl_4_	Cs_4_Pb_0.95_Mn_0.05_Br_1.9_Cl_4.1_	Cs_4_Pb_0.9_Mn_0.1_Br_1.8_Cl_4.2_	Cs_4_Pb_0.8_Mn_0.2_Br_1.6_Cl_4.4_
Wt. fraction	87%	94%	100%	92%
*a*/Å	13.3555(2)	13.3465(1)	13.3010(1)	13.2653(2)
*c*/Å	16.8135(4)	16.8248(3)	16.7800(3)	16.7471(3)
*V*/Å^3^	2597.22(6)	2595.47(5)	2570.94(5)	2552.13(5)
*Z*	6	6	6	6
**Formula**	**Cs**_**4**_ **Pb(Br/Cl)**_**6**_	**5% Mn**	**10% Mn**	**20% Mn**
Space group	*Pm* $\bar 3$ *m*	*Pm* $\bar 3$ *m*	—	*Pm* $\bar 3$ *m*
Composition	3D CsPbBr_3_	3D CsPbBr_3_	—	CsCl
Wt. fraction	13%	6%	—	8%
*a*/Å	5.6421(19)	5.6421(19)	—	4.126
*V*/Å^3^	176.61(18)	176.61(18)	—	70.24
*Z*	1	1	1	1
*Χ* ^2^	2.70	2.65	2.67	2.96
*R*_wp_ (%)	5.59	5.45	5.47	5.68
*R*_p_ (%)	4.14	4.12	4.08	4.31

The number in the parentheses are the estimated standard deviation of the last significant figure

### Phase stabilization of Cs_4_PbX_6_

The versatility of the Cs–Pb–X ternary compounds (particularly Cs_4_PbX_6_ and CsPbX_3_) lies in their ability to interconversion via post-synthetic physical and chemical treatments^29^. The post-synthetic conversion of cubic CsPbX_3_ to rhombohedral Cs_4_PbX_6_ has been demonstrated via amine- and thiol-mediated extraction of PbBr_2_^30^, while the reverse conversion from Cs_4_PbX_6_ to CsPbX_3_ has been reported by the insertion of PbX_2_, stripping of CsX, excess oleic acid, and by heat treatment (90 to 180 °C)^18^. These post-synthetic conversion mechanisms are inadequate to explain the simultaneous formation of Cs_4_PbX_6_ and CsPbX_3_ phases, during synthesis. It has been demonstrated that the phase stabilization of perovskite semiconductors could be achieved by controlling the octahedral tilting via partially substituting Pb^2+^ with other metal ions. For instance, substitution of smaller Mn^2+^ stabilizes the cubic α-CsPbI_3_ phase by reducing the octahedral rotation or tilting via a decrease in the bond angle of Pb–I–Pb below 180°, which is along the interconnected neighboring PbX_6_ octahedra through the bridging halide ion^31^. In the present case, a similar octahedral tilting (enhanced) results in the phase stabilization of low-symmetry Cs_4_Pb(Br/Cl)_6_ upon Mn^2+^ incorporation. A decrease in the Pb–X bond length after Mn doping (10% Mn) to 2.950 Å, than undoped Cs_4_Pb(Br/Cl)_6_ (2.991 Å), resulted in an enhanced octahedral tilting, preventing the undesired high-symmetry cubic CsPbX_3_ phase (Fig. 1c). Here, the octahedral tilting in the Cs_4_PbX_6_ (X = Br/Cl) is described based on the X–Pb–Cs_1_ bond angle along *c* axis of the unit cell, which changes from 54.94° (undoped) to 54.76° upon Mn incorporation. This octahedral tilting would distort the PbX_6_ octahedra, which subsequently lowers the symmetry of crystal structure. It is worth mentioning that only two stable compositions exist in the phase diagram of mixed CsX-PbX_2_, under Cs-rich or Pb-deficient conditions viz., low-symmetry rhombohedral Cs_4_PbX_6_ and high-symmetry cubic CsPbX_3_^32^. Therefore, enhanced octahedral tilting upon Mn substitution, result in the formation of only possible low-symmetry Cs_4_PbX_6_ phase; at the same time, destabilizing the cubic CsPbX_3_ phase under Cs-rich condition. This phase stabilization of 0D Cs_4_PbX_6_ via Mn substitution, might favor deeper investigation to their unexplored and intriguing properties.

### Electronic structure

The changes in the electronic structure namely bonding nature and electron density distribution in the Cs_4_PbCl_6_ were analyzed with Mn incorporation via maximum entropy method (MEM) using XRD results. In the Cs_4_PbCl_6_, the electron density around Pb and Cl is anisotropic and highly resolved with no feature of electron sharing between Pb and Cl, resembling electrostatic attractive/repulsive forces (Fig. 1d). Upon Mn substitution a significant change in the local charge density around Pb atom was apparent, suggesting that Mn modulates the electronic structure of the 0D Cs_4_PbCl_6_ (Fig. 1e, f). With increasing Mn content, high electron density distribution between Pb and Cl atoms, suggests covalent bonding nature of (Pb/Mn)–Cl bonds (Supplementary Figure 6). This also supports the stronger (Pb/Mn)–Cl bonds or shorter bond lengths of Pb–Cl upon Mn doping, as observed from XRD. The change in local electron density after Mn-doping results in the lattice distortion (also evidenced from octahedral tilting, Fig. 1c) and could lead to electron-phonon (lattice) coupling and formation of polaron^33^. Recently, the formation of polarons in the Cs_4_PbBr_6_ has been reported from density functional theory calculation and transient absorption measurements^10^. In the present case, at variance with the undoped Cs_4_PbX_6_, a strong lattice distortion upon Mn incorporation could eventually enhance the electron-phonon coupling, leading to polaron formation. Therefore, it could be concluded that Mn^2+^ incorporation modulates the electronic structure and favors the phase stabilization of Cs_4_PbX_6_, preventing the formation of undesirable CsPbX_3_ impurity (Fig. 1g). Though the phase stabilization of Cs_4_PbX_6_ were concluded based on XRD results, this technique has its own limitation of insensitivity to detect any minor crystalline phase below 5%^34^. Henceforth, to further support the phase stabilization, optical characterization was exploited owing to its high sensitivity to detect even trace of fluorescent CsPbX_3_ impurity.

**Fig. 2:**
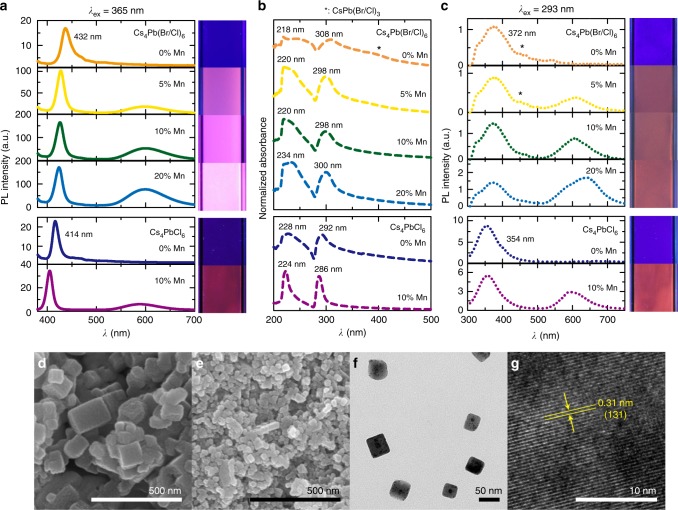
Luminescent properties of Mn^2+^-doped Cs_4_PbX_6_. **a** PL spectra of Mn^2+^-doped Cs_4_PbX_6_ colloids were measured under 365 nm excitation, where X = Br/Cl and Cl, with Mn contents denoted as 0% Mn, 5% Mn, 10% Mn, and 20% Mn and the insets shows the respective samples under 365 nm UV lamp. **b** UV-Vis spectra of Mn^2+^-doped Cs_4_PbX_6_ colloids. **c** PL spectra of Mn^2+^-doped Cs_4_PbX_6_ colloids were measured under 290 nm excitation and the insets shows the respective samples under 254 nm UV lamp. The PL spectra presented are the PL intensities corrected using optical density. The asterix mark denote the 3D CsPb(Br/Cl)_3_ phases. FE-SEM images of **d** undoped Cs_4_Pb(Br/Cl)_6_ and **e** Cs_4_Pb(Br/Cl)_6_:10% Mn, and **f**, **g** HR-TEM images of Cs_4_Pb(Br/Cl)_6_:10% Mn. A lattice spacing of 0.31 nm corresponding to (131) plane of rhombohedral structure of Cs_4_Pb(Br/Cl)_6_

The excitation and photoluminescence (PL) properties of Cs_4_PbX_6_:Mn colloids were presented in Fig. 2. The undoped Cs_4_Pb(Br/Cl)_6_ exhibited broad emission at 432 nm (ranging 415 to 470 nm) under 365 nm excitation (Fig. 2a). The broad emission arises from the contribution from “D-states” of the host and 3D CsPb(Br/Cl)_3_ impurity (443 nm emission, Supplementary Figure 7)_,_ where the former dominates the latter. The presence of CsPb(Br/Cl_3_) impurity was also evident from the broad absorption ranging 360 to 400 nm in the undoped sample (Fig. 2b). It is to be noted that D-state emission arise from the charge transfer from halide ions of the PbX_6_ octahedra to Pb^2+^ ion occupying mismatched neighboring Cs^+^ sites, resulting in the creation of Frenkel excitons localized at the vicinity of Pb ions^19,35^. Upon Mn incorporation (5% Mn), a narrow emission band at 420 nm was observed, corresponding to the D-state of Cs_4_Pb(Br/Cl)_6_ phase, with feeble emission from CsPb(Br/Cl)_3_ impurity. The inclusion of chlorine during Mn doping (MnCl_2_ precursor) may contribute meagerly to the D-state emission compared to undoped one. Further increase in Mn content (above 10% Mn), eliminated the CsPb(Br/Cl)_3_ impurity as evidenced from its negligible absorption (360–400 nm), absorption narrowing, and unaltered D-state emission (Fig. 2a, b). The above results reaffirm the strong phase stabilization of Cs_4_PbX_6_ in presence of Mn, further corroborating the XRD results (Fig. 1a)^36,37^. The additional orange emission at 599 nm, is assigned to the forbidden Mn^2+^ *d*–*d* transitions^22^, indicating the presence of exchange coupling between charge carriers of Cs_4_Pb(Br/Cl)_6_ and Mn^2+^ ions. Further, the single-halide Cs_4_PbX_6_ analogs were investigated to understand the role of Mn^2+^ on the optoelectronic properties and distinguish them from halide effect, which is a limitation in the mixed halides. The undoped Cs_4_PbBr_6_ exhibited two UV emission bands and intense green emission (512 nm) arising from Pb^2+^/D-state emission and CsPbBr_3_ impurity, respectively (Supplementary Figure 8 and Supplementary Note 5). While in the undoped Cs_4_PbCl_6_, the 414 nm emission corresponds to the band-edge emission of CsPbCl_3_ impurity^12^ and the hump ranging 450 to 480 nm is assigned to the quantum size effect of CsPbCl_3_. Upon Mn incorporation, a new emission at 400 nm arises from the D-state of Cs_4_PbCl_6_, with less contribution from CsPbCl_3_, compared to undoped one, which completely disappeared with higher Mn (20% Mn) content (Supplementary Figure 4), reaffirming the phase stabilization of Cs_4_PbX_6_ upon Mn incorporation.

The undoped and Mn^2+^-doped Cs_4_PbCl_6_ displayed blue and pinkish (combination of D-state ‘blue’ and Mn^2+^ ‘orange’) emission under 365 nm, respectively (Fig. 2a, inset). The dual UV absorption bands at 218 and 308 nm, are the characteristic features of Cs_4_Pb(Br/Cl)_6_. The band gaps estimated from the absorption maxima are 3.83, 4.02, and 4.25 eV, for Cs_4_PbBr_6_, Cs_4_Pb(Br/Cl)_6,_ and Cs_4_PbCl_6_, respectively (Supplementary Figure 8a), which agrees with the earlier reports^18^. It is interesting to note the increase in Cs_4_PbX_6_ host emission after Mn doping (Fig. 2a–c) in both mixed halide and chloride analog, similar to the perovskite nanocrystals^38^. The host emission increment is more prominent in the mixed halide (Br/Cl) analog than pure chloride, which is presumed to the passivation effect of chloride ion, on the pre-existing defects of the host^38^. Therefore, Mn^2+^ doping significantly alters the competitive kinetics between the radiative and non-radiative relaxation process of the Cs_4_PbX_6_ host.

The PL properties were investigated, by exciting at energies higher than band gap of host (290 nm), due to their strong absorption. Mn^2+^-doped (Br/Cl) samples exhibited dual UV emissions at 372 nm and a hump at 324 nm, arising from optical ^3^P_0,1,2_ → ^1^S_0_ transitions of Pb^2+^ ion^35,39^ and henceforth denoted as ‘Pb^2+^ ion’ emission (Fig. 2c) . Unlike at 365 nm excitation, the D-state (420 nm) emission of both undoped and Mn^2+^-doped was either absent or buried in the broad 300 to 450 nm spectral range under 290 nm excitation. A similar, broad Pb^2+^ emission (354 nm) was observed for Cs_4_PbCl_6_^12^, without the D-state emission, when excited at 290 nm. With Mn doping, an apparent drop in the 3D CsPb(Br/Cl)_3_ emission (ranging 430 to 450 nm) further demonstrates the phase stabilization of Cs_4_PbX_6_ (Fig. 2c). In addition, despite the unaltered Pb^2+^ emission, an enhanced Mn^2+^ emission in the mixed halides, was observed with increasing Mn concentration, due to efficient energy transfer from Pb^2+^ to Mn^2+^. The unusual red-shifted emission from 599 to 635 nm for Cs_4_Pb(Br/Cl)_6_:20% Mn sample, is attributed to the formation of Mn-to-Mn pairs at higher Mn concentration, which reduces the ^4^T_1_ - ^6^A_1_ energy gap_,_ resulting in the red shift of Mn^2+^ emission^40^. Hence, the phase stabilization of Cs_4_PbX_6_ (X = Cl, Br/Cl) with Mn incorporation were demonstrated in the chlorine-dominant system (above 60%), through XRD and optical results.

The Mn incorporation was also investigated in bromide analog (Cs_4_PbBr_6_) for monitoring phase stabilization via XRD and PL results (Supplementary Figures 2 and 8). With increasing Mn, the green emission (512 nm) of CsPbBr_3_ impurity decreased gradually, which completely disappeared above 70% Mn, suggesting the prevention of CsPbBr_3_ impurity in presence of Mn, agreeing with the XRD results. However, unlike Br/Cl and Cl analogs, the bromide counterpart necessitates higher Mn^2+^ concentration (above 50% Mn) to stabilize the Cs_4_PbBr_6_ structure, which is ascribed to the low substitution ratio of Mn^2+^ observed in the bromine-dominated Cs–Pb–Br perovskite-like system^41^. In addition, this leads to residual segregation of CsBr, at higher Mn concentration (above 50% Mn). The above results further validate the phase stabilization of zero-dimensional Cs_4_PbX_6_ upon Mn incorporation in all the bromine, Br/Cl, and chlorine analogs.

The synthesized Cs_4_Pb(Br/Cl)_6_:10% Mn exhibited cubic morphology with particle size ranging between 20 and 40 nm (Fig. 2d–g) which is much smaller than undoped one (50 to 120 nm). Further increasing the Mn content did not affect the particle size. It is worthy to note that the emission property of Cs_4_PbX_6_ host is independent of the particle size due to its intrinsic structural confinement and extremely localized emissive centers (isolated octahedra). However, the dependence of size and morphology of Cs_4_PbX_6_ on the length of alkyl chains of amine surfactant were clearly apparent. Unlike long-chain amines (oleylamine), which lead to a narrow particle size distribution in the 26 ± 4 nm range (mixture of spherical, hexagonal, and cubic morphologies)^20^, the use of shorter-chain octylamine in the present work favored a cubic shape with a relatively wide size distribution.

### Probing the host emissive states

**Fig. 3:**
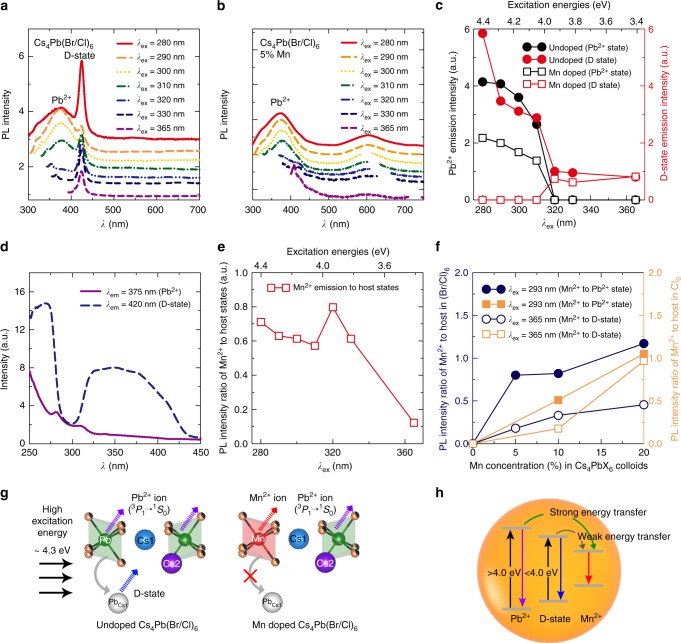
Probing the host states at the isolated octahedron with Mn^2+^ ion. Colloidal PL spectra measured at different excitation wavelengths ranging 280 to 365 nm for the **a** undoped Cs_4_Pb(Br/Cl)_6_ and **b** Cs_4_Pb(Br/Cl)_6_:5% Mn. **c** Emission intensities of Pb^2+^ and D-state emission with varying excitation energies for the undoped and Mn-doped Cs_4_Pb(Br/Cl)_6_:5% Mn, **d** excitation spectra of Cs_4_Pb(Br/Cl)_6_:5% Mn colloids by monitoring at 375 nm (Pb^2+^ ion), and 420 nm (D-state), **e** PL intensity ratio of Mn^2+^ to the host emission with respect to varying excitation energies, **f** PL intensity ratio of Mn^2+^ to the host states with varying Mn concentration for Cs_4_PbX_6_ colloids, where X = (Br/Cl) and Cl, **g** origin of host emissions namely Pb^2+^ and D-state under high excitation energies, without and with Mn doping at high excitation energies. The solid, dashed, and curved arrows are denoted for optical excitation, emission, and energy transfer processes, respectively. **h** Strength of energy transfer from the host states namely Pb^2+^ and D-state to the Mn^2+^ emission are depicted. The upward, downward, and curved arrows are denoted for the optical excitation, emission, and energy transfer processes, respectively

The excitation-dependent PL properties of Cs_4_Pb(Br/Cl)_6_ were explored to get insight of multiple luminescent states of the host. A range of excitation wavelengths from 280 to 365 nm (4.40 to 3.40 eV) were employed including excitation energies higher than the band gap of Cs_4_Pb(Br/Cl)_6_ host (4.00 eV). At higher excitation energies (*λ*_ex_ of 280 nm), dominant D-state emission (432 nm) was accompanied by Pb^2+^ emission (324 and 375 nm) for undoped Cs_4_Pb(Br/Cl)_6_ (Fig. 3a). It is to be noted that 432 nm emission is denoted here as D-state emission, disregarding the less contribution from CsPb(Br/Cl)_3_ impurity (Fig. 2a), for the ease of emphasizing on dependence of host emissions (Pb^2+^ and D-state) with varying excitation energies. As excitation wavelength increased from 280 to 310 nm, the D-state emission gradually decreased, where Pb^2+^ emission remains dominant. While above 310 nm excitation, Pb^2+^ emission disappeared due to insufficient energy to excite its ^3^P_1_ levels and only D-state is retained. Upon Mn doping, the D-state emission completely disappeared in the excitation wavelengths ranging, 280 to 310 nm, exhibiting only Pb^2+^ emission (Fig. 3b, c). The absence of D-state emission after Mn^2+^ doping is probably due to suppression of charge-transfer process from PbX_6_ octahedra to Pb^2+^ occupying Cs^+^ site, primarily at high excitation energies (above 4.0 eV), where both Pb^2+^ and D-states are excited. Therefore, the presence of Mn^2+^ plays a key role in regulating the optical behavior of molecule-like isolated PbX_6_ octahedra in Cs_4_PbX_6_ nanocrystals.

The energy transfer and direct excitation of ^3^P_1_ levels of Pb^2+^ (375 nm) and D-states (420 nm), were explored from excitation spectrum of 5% Mn^2+^-doped Cs_4_Pb(Br/Cl)_6_ (Fig. 3d). The spectrum reveals Pb^2+^ ion can be excited at high excitation energies above the band gap of Cs_4_Pb(Br/Cl)_6_ host (below 310 nm excitation); while the D-state is excited at lower excitation energies typically below the host band gap, (above 310 nm excitation). A direct excitation of D-state at 280 nm, is also apparent along with Pb^2+^ ion^35^. However, the energy transfer between the Pb^2+^ and D-state of the host cannot be ruled out for the undoped sample, due to the occurrence of D-state emission in the excitation wavelengths (290 to 310 nm) where only Pb^2+^ state is excited, as reported earlier^35^, and is consistent with Cs_4_PbX_6_ solids (Supplementary Figure 9 and Supplementary Note 6).

Moreover, Mn^2+^ emission is well-known to be sensitized by the host via energy transfer, where Pb^2+^ and D-state sensitizes at high and low excitation energies, respectively. Therefore, Mn^2+^ emission at varying excitation energies is determined from the absorption of the corresponding host state and efficiency of energy transfer from host states. The Mn^2+^ emission relative to the total host emission (i.e., Pb^2+^ + D-state) was the highest at 280 nm, attributing to the efficient energy transfer from the Pb^2+^ state, which then gradually decreased at lower excitation energies, apart from 320 nm (Fig. 3e). Although the direct excitation of both Pb^2+^ and D-states is possible at 280 nm, Mn^2+^ is sensitized only through Pb^2+^ state, owing to the suppression of D-state in presence of Mn. The, dominant Mn^2+^ emission at 320 nm excitation, ascribed to the Mn^2+^ sensitization from both D-state and partially from Pb^2+^ ion. In addition, energy transfer from Pb^2+^ ion to Mn^2+^ was more efficient than from D-state (Fig. 3f, Supplementary Figure 10, and Supplementary Note 7). Moreover, the presence of Mn^2+^ considerably suppresses the D-state emission by blocking the charge-transfer process from isolated octahedra to the D-state, especially at high excitation energies (about 4.3 eV, 280 nm excitation), where both the D-state and Pb^2+^ could be directly excited (Fig. 3g). This may be caused by the weak coupling between electronically decoupled (Pb/Mn)X_6_ octahedra and D-state (Pb^2+^ at Cs site) which deteriorates the charge transfer between them, due to strong Mn–Cl bond formation. The sensitization of Mn^2+^ is predominantly occurs from Pb^2+^ than the D-state, due to larger energy difference between Mn^2+^ and Pb^2+^ states (Fig. 3h). Moreover, the efficient energy transfer to Mn^2+^ dopant in the wide band gap host, due to large energy difference between host and Mn^2+^ levels, is well documented^38^. The above results demonstrate that the Mn^2+^ incorporation significantly affects the optical properties of host state in Cs_4_PbX_6_ nanocrystals.

### Luminescence efficiency

The potential application of perovskite-like Cs_4_PbX_6_ as emitting layer in color-converting devices has been explored in colloidal and solid forms (thin films and powders)^16^ and is expected to have high conversion efficiency similar to other color converter viz. phosphor^42^. High PL QY has been reported in perovskite colloids by employing ligands to stabilize the nanocrystals^1,43^, and thin films (solids) of micron-sized grains^44,45^. Here, although PL QY of undoped Cs_4_Pb(Br/Cl)_6_ nanocrystal is low (5%), the overall QY substantially increased to 32% upon Mn^2+^ doping, under 365 nm excitation (Table 2, Supplementary Figure 11a, and Supplementary Note 8). Highest PL QY of 29% was obtained for Cs_4_Pb(Br/Cl)_6_:10% Mn and Cs_4_PbCl_6_:10% Mn (Table 2), which is among the best values reported for Mn^2+^ emission (Supplementary Table 8)^46^. In addition, Mn^2+^ luminescence was the highest at lower (365 nm) and higher excitation energy (290 nm), for Cs_4_Pb(Br/Cl)_6_ and Cs_4_PbCl_6_, respectively, due to their suitable band gaps (determined by halide species), enabling efficient energy transfer to Mn^2+^. The overall superior PL QY of Mn^2+^-doped Cs_4_PbX_6_ colloids (X = Br/Cl, Cl) is attributed to the spatially confined zero-dimensional structure, and presence of dopant at the isolated octahedra favoring enhanced dopant emission through the localized excitons and high exciton binding energy (Supplementary Figure 11b to 11e). The reported exciton binding energies of Cs_4_PbBr_6_ and Cs_4_PbCl_6_ are 353^3^ and 153 eV^47^, respectively, compared to CsPbBr_3_ (19 to 62 meV) and CsPbCl_3_ (72 meV)^48–50^. These results suggest that the high luminescent Cs_4_PbX_6_ nanocrystals doped with Mn^2+^ could represent promising emitting materials for efficient solid-state lighting applications.

**Table 2:** PL quantum yield of the Cs_4_PbX_6_ colloids in n-octane medium

Sample	*λ*_ex_ (nm)	total QY (%)	QY (%)	
			Host emission (%)	Mn^2+^ emission (%)
Cs_4_PbBr_6_	365	60%	60	0
Cs_4_Pb(Br/Cl)_6_	365	5%	5	0
Cs_4_Pb(Br/Cl)_6_:5% Mn	365	32%	5	27
Cs_4_Pb(Br/Cl)_6_:10% Mn	365	32%	3	29
Cs_4_Pb(Br/Cl)_6_:20% Mn	365	23%	2	21
Cs_4_PbCl_6_	290	6%	6	0
Cs_4_PbCl_6_:10% Mn	290	32%	5	27
Cs_4_PbCl_6_	365	2%	2	0
Cs_4_PbCl_6_:10% Mn	365	15%	6	9

**Fig. 4:**
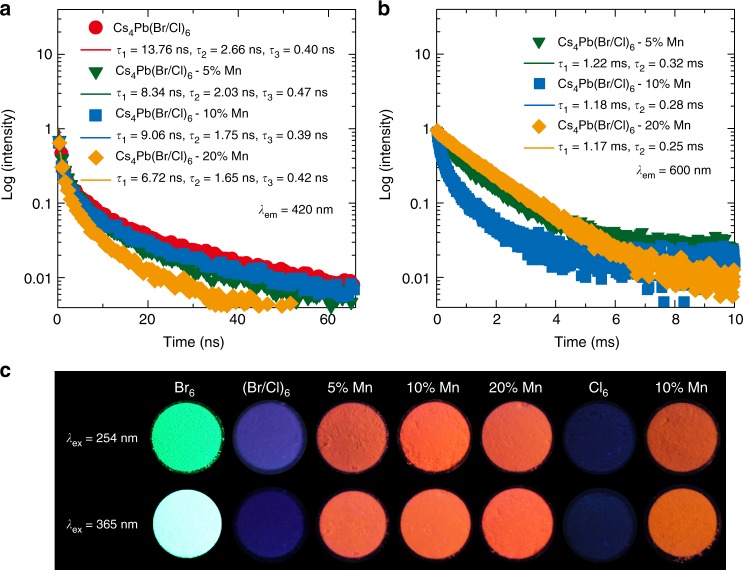
Lifetime of Mn^2+^-doped Cs_4_PbX_6_. Time-resolved PL spectra of Cs_4_Pb(Br/Cl)_6_ colloids excited at 375 nm and monitoring the D-state and Mn^2+^ emission at **a** 420 and **b** 600 nm, respectively. **c** Images of Cs_4_PbX_6_ perovskite solids under 254 and 365 nm UV lamp, which are denoted by their respective halides and Mn concentrations

In the Cs_4_PbX_6_ solids, a similar low PL QY was observed for undoped sample, but the overall PL QY increased markedly upon Mn doping in both mixed halide and chloride samples (Supplementary Figure 11e). PL QY of 21% was obtained for Mn^2+^ emission in Cs_4_PbCl_6_ solids, under 290 nm excitation (Supplementary Table 9). The bright orange emission of Mn^2+^-doped Cs_4_PbX_6_ in their solid form, is a unique feature among Mn^2+^-doped perovskite solids (Fig. 4c). This remarkable PL QY and retention of dopant emission in solid form is the attributes of spatial confinement of low-dimensional Cs_4_PbX_6_ induced by isolated octahedra, eventually favoring a stable dopant emission in solids.

To further elucidate the emission properties of Cs_4_PbX_6_ host and Mn^2+^, time-resolved PL results were performed by monitoring the D-state (420 nm) and Mn^2+^ emission (600 nm), exciting at 375 nm (Fig. 4a, b, Supplementary Figures 12 to 14, and Supplementary Note 9). A nominal decrease in the average lifetime of D-state emission in Cs_4_PbX_6_ (X = Br/Cl and Cl) was observed with increasing Mn content (Supplementary Figure 13). For instance, the average lifetime of D-state decreased from 1.08 ns (undoped) to 0.82 ns (20% Mn), corroborating the Mn^2+^ sensitization by the Cs_4_Pb(Br_/_Cl)_6_ host (Table 3). A long lifetime was obtained for the Mn^2+^ emission in Cs_4_Pb(Br/Cl)_6_ and Cs_4_PbCl_6_ of 1.20 ms and 1.39 ms, respectively (Fig. 4b and Supplementary Figure 13b). The lifetime of D-states of Cs_4_Pb(Br/Cl)_6_ converged with three components namely 3.7 (slow), 2.6, and 0.40 ns (last two combined is fast), similar to the previous report^3^. The first slow (radiative) component is assigned to D-state emission and the fast (non-radiative) component to the energy transfer process to Mn^2+^. A rise in the contribution of slow component with increasing Mn^2+^ content, agrees with the increase in D-state emission (Supplementary Figure 12). This suggests that the inclusion of chlorine species with Mn-doping (MnCl_2_ precursor), passivates the pre-existing defects in the Cs_4_Pb(Br/Cl)_6_ host, resulting to an enhanced host PL efficiency^38^. Hence, the passivation effect of chlorine in the Cs_4_Pb(Br/Cl)_6_, would contribute greatly to the PL efficiency of D-state rather than energy transfer to Mn^2+^ at 375 nm excitation. On the contrary, superior contribution of fast component in Cs_4_PbCl_6_, illustrates the dominant energy transfer from D-state to Mn^2+^ (Supplementary Figures 13 and 14) at 375 nm excitation. The chloride passivation effect in the Cs_4_PbCl_6_ may be negligible as chloride content remain unchanged with increasing dopant concentration. Therefore, the D-state emission in mixed halide Cs_4_Pb(Br/Cl)_6_, is predominated by the passivation effect of chlorine over energy transfer to Mn^2+^, while in the Cs_4_PbCl_6_, energy transfer to Mn^2+^ dominated over passivation effect.

**Table 3:** Average lifetime of D-state emission of Cs_4_Pb(Br/Cl)_6_ and Cs_4_PbCl_6_ monitored at 420 nm and 400 nm, respectively

Mn content (%)	Average lifetime of D-state emission (ns)	
	Cs_4_Pb(Br/Cl)_6_	Cs_4_PbCl_6_
0	1.08	1.47
5	1.00	0.44
10	0.85	2.50
20	0.82	0.32

**Fig. 5:**
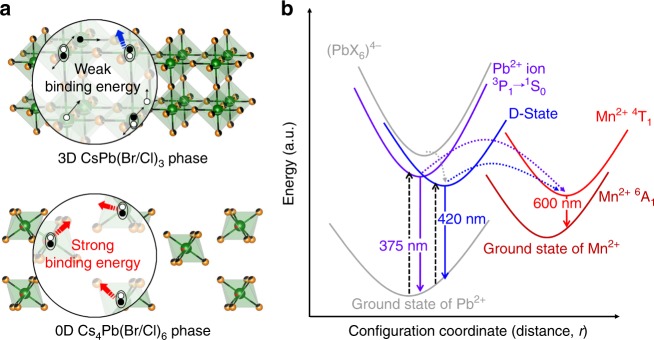
Mechanism of energy transfer from host states to Mn^2+^. **a** Illustration of high exciton binding energy (at above 150 meV) due to the localized excitons formed in the isolated octahedra of 0D Cs_4_PbX_6_ (X = Cl, Br/Cl) perovskite, compared to low binding energies (less than 60 meV) of 3D CsPbX_3_ perovskite, where filled and hollow circles represent electrons and holes, respectively. **b** Configurational coordinate representation of Cs_4_Pb(Br/Cl)_6_ perovskite depicting Pb^2+^-based (^3^P_0__,__1_  →^1^S_0_ transition) and D-state emissions, and their energy transfer to the Mn^2+^ energy levels. The long dash and dotted arrows depicts the excitation and energy transfer process leading to emission (solid arrows)

The mechanism of energy transfer from host states (Pb^2+^ and D-state) to Mn^2+^ in the mixed halide perovskite is further illustrated in the Fig. 5. The high PL QY of Mn^2+^-doped Cs_4_PbX_6_ is the attributes of structure-induced quantum confinement with high exciton binding energy favoring enhanced emission, compared to 3D CsPbX_3_ perovskite (Fig. 5a). The superior PL QY of Mn^2+^-doped Cs_4_Pb(Br/Cl)_6_ and Cs_4_PbCl_6_ were observed at low (365 nm) and high excitation energy (290 nm), due to high absorption in their respective band gaps arising from halide identity. The Pb^2+^ ions are excited at high excitation energies (above 4.0 eV), leading to 375 nm emission, while D-states are excited at low excitation energies (below 4.0 eV), and both these states eventually generate Mn^2+^ emission through energy transfer (Fig. 5b). The presence of Mn^2+^ suppresses the charge transfer from isolated octahedra to the D-state, due to the weak coupling with the regular crystal lattice sites particularly at higher excitation energy. The enhanced overall PL QY of Mn^2+^-doped Cs_4_PbX_6_ in both colloidal and solid form is attributed to the synergistic effect of structural quantum confinement effect from the isolated octahedra and high exciton binding energies, facilitating dopant emission through enhanced energy transfer process.

## Discussion

In conclusion, we rationalized the exceptional optical behavior of isolated octahedra in the zero-dimensional cesium lead halide structure by introducing Mn^2+^ dopants. Structural, PL, and lifetime results confirmed the incorporation of Mn^2+^ in the 0D Cs_4_PbX_6_ lattice. The Mn^2+^ incorporation stabilized the Cs_4_PbX_6_ structure via enhanced octahedral tilting and limited compositional variation of Cs–Pb salts, suppressing the formation of CsPbX_3_ impurity phase. The PL QY at a given excitation energy was determined by the energy transfer from the host states to Mn^2+^ and band gap of the host. A high PL QY for Mn^2+^ emission was achieved in the colloidal (29%) and solid (21%, powder) forms; making the solid form of Cs_4_PbX_6_ as a promising emissive layer for light-emitting devices. The enhanced PL QY of the Mn^2+^ emission was attributed to the synergistic effect of structure-induced spatial confinement of 0D Cs_4_PbX_6_ and electronically decoupled PbX_6_ octahedra favoring dopant emission via localized exciton. The present work provides deep insight into the structure and molecular behavior of 0D perovskite-like Cs_4_PbX_6_ material and opens an avenue to design low-dimensional perovskites with photo and chemical stability for high-performance optoelectronic applications.

## Methods

### Materials

All the reagents were used without any purification. Cesium carbonate (Cs_2_CO_3_, 99.999% Kojundo), lead (II) bromide (PbBr_2_, 99%, Sigma-Aldrich), lead (II) chloride (PbCl_2_, 98%, Sigma-Aldrich), manganese (II) bromide (MnBr_2_, 98%, Sigma-Aldrich) manganese (II) chloride tetrahydrate (MnCl_2_.4H_2_O, 99%, Sigma-Aldrich), oleic acid (OA, 90%, Fluka), octylamine (OcA, 97%, Sigma-Aldrich), N,N-dimethylformamide (DMF, 99.8%, Sigma-Aldrich), n-hexane (99.8%, Junsei, Japan), n-octane (90%, Sigma-Aldrich), 1-octadecene (95%, Sigma-Aldrich), hydrochloric acid (37%, Sigma-Aldrich), and hydrobromic acid (48%, Sigma-Aldrich) were employed for the synthesis.

### Synthesis of undoped and Mn^2+^-doped Cs_4_Pb(Br/Cl)_6_ colloids

The exact compositions of the synthesized undoped and Mn^2+^-doped Cs_4_Pb(Br/Cl)_6_ series are Cs_4_PbBr_2_Cl_4_ and Cs_4_Pb_1__–__*x*_Mn_*x*_Br_2–2*x*_Cl_4+2*x*_ with *x* = 0.05 (5% Mn), 0.10 (10% Mn), and 0.20 (20% Mn), respectively. Mn^2+^-doped Cs_4_Pb(Br/Cl)_6_ nanocrystals were synthesized by a reverse microemulsion method at room temperature, as reported earlier^20^. PbBr_2_ precursor was used as the lead precursor. A simultaneous anion (Cl^–^) and cation (Mn^2+^) inclusion were effected by employing MnCl_2_.4H_2_O precursor for the synthesis of Mn^2+^-doped Cs_4_Pb(Br/Cl)_6_. An optimal concentration of octylamine (OcA) and oleic acid (OA) surfactants were used in facilitating the solubilization of Cs, Pb, and Mn precursors. The mixed MnCl_2_.4H_2_O and PbBr_2_ precursors, and the Cs-oleate precursor were synthesized separately. First, a mixture of 2.25 g of Cs_2_CO_3_ and 21.5 mL of OA was stirred and degassed at 130 °C, which is further maintained under vacuum for 1 h to generate a yellowish stock of Cs-oleate precursor. Second, 0.2 mL Cs-oleate precursor, 10 mL of n-hexane, 5 mL of OA were loaded into a 50-mL three-neck flask, followed by mild degassing (vacuum) for 5 min and Argon purging for 5 min. Third, a mixture of PbBr_2_ and MnCl_2_.4H_2_O (DMF, 1 mL), HCl (38 wt%, 19 μL), 0.10 mL OA, and 0.05 mL OcA was swiftly injected into the flask, under vigorous stirring. Upon stirring for 10 min, white color crystals were formed suggesting the formation of Mn^2+^-doped Cs_4_Pb(Br/Cl)_6._ The as-synthesized nanocrystals were collected via centrifugation at 7000 rpm for 5 min followed by dispersion in 2 mL of n-octane for further characterization. The contents of Cs-oleate, HCl, and surfactants (OA, OcA) were maintained the same for varying concentration of Mn precursors.

The undoped Cs_4_Pb(Br/Cl)_6_ was synthesized following the same procedure, in the absence of MnCl_2_.4H_2_O. A color change from a pale-white to green was observed in 10 min upon the addition of Pb precursor into the Cs-oleate, which is then centrifuged and dispersed in n-octane for further characterization. N-octane was employed as the solvent for the Mn^2+^-doped colloids for its superior dispersibility over hexane and toluene.

### Synthesis of undoped and Mn^2+^-doped Cs_4_PbX_6_ (X = Br, Cl)

Cs_4_PbX_6_ samples were synthesized using stoichiometric quantity of PbX_2_ and HX (15 μL) as the lead and halide precursors along with Cs-oleate, where X = Br, Cl. The limited solubility of PbCl_2_ in DMF solvent at room temperature was overcome by warming the contents until it gets dissolved. Mn^2+^-doped Cs_4_PbX_6_ samples were synthesized following the same procedure along with manganese precursors of MnCl_2_.4H_2_O and MnBr_2_, for the chloride and bromide analog, respectively. 

### Synthesis of CsPbX_3_ (X = Br, Br/Cl, Cl) perovskites

CsPbX_3_ (X = Br, Br/Cl, Cl) nanocrystals were synthesized as described by Palazon et al.^29^. In a typical synthesis, PbX_2_ (0.2 mmol; X = Br, Cl, Br/Cl), 0.47 mL oleic acid, 5.0 mL octadecene, and 0.50 mL oleylamine were loaded in a 25 mL 3-neck flask and dried under vacuum at 100 °C for 15 min. The temperature was raised to 190 °C, after degassing and 0.5 mL of Cs-oleate solution (made from 0.4 g of Cs_2_CO_3_ dissolved in 11.75 g of octadecene, and 1.55 g oleic acid at 100 °C under vacuum), which was pre-heated on a hot-plate at 100 °C was swiftly injected. Five seconds after the injection, the nanocrystal solution was quickly cooled to room temperature with an ice bath and by the addition of 5 mL of toluene (at 100 °C). The nanocrystals were collected by centrifugation (2500 rpm/2 min.) and re-dispersed in 5 mL toluene. The composition of (Br/Cl) analog is CsPb(Br_0.4_/Cl_0.6_)_3_, where a mix of 0.08 mmol of PbBr_2_ and 0.12 mmol of PbCl_2_ precursor were employed for the synthesis.

### Synthesis of zero-dimensional Cs_4_PbX_6_ solids

The as obtained colloidal samples dispersed in n-octane were centrifuged and re-dispersed in tert-butanol and centrifuged at 7000 rpm for 5 min. The samples were then dried at 80 °C overnight to get respective Cs_4_PbX_6_  solids. The body color of the zero-dimensional solids were pure white for the Cs_4_PbCl_6_, Cs_4_PbCl_6_:Mn, and Cs_4_Pb(Br/Cl)_6_:Mn_,_ except Cs_4_Pb(Br/Cl)_6_ (pale greenish white), and Cs_4_PbBr_6_ (bright yellow).

### Characterization

The synthesized zero-dimensional Cs_4_PbX_6_ nanocrystals were characterized by powder X-ray diffraction (XRD), X-ray photoelectron spectroscopy (XPS), electron spin resonance spectra (ESR), inductive coupled plasma-optical emission spectroscopy (ICP-OES), field-emission scanning electron microscope (FE-SEM), and high-resolution tunneling electron microscope (HR-TEM). The XRD was performed using a Philips X’Pert diffractometer with Cu Kα radiation, in the range of 10° to 120° with a step size of 0.026° with the synthesized powders. The structural information was derived from Rietveld refinement using the General Structure Analysis System (GSAS) software suite. The VESTA program was used to draw the crystal structure^51^. The phase fractions of multiple phases were estimated using Rietveld refinement of XRD results considering full refinement of crystallographic and instrumental parameters as implemented in the GSAS program suite. The FE-SEM and HR-TEM images were recorded using a Hitachi S-4700 and FEI Tecnai F20 (200 kV), respectively, at Korea Basic Science Institute (KBSI), Gwangju, South Korea. The sample for TEM measurements were prepared by dispersing the nanocrystals in the ethanol medium. It is to be noted that the undoped and Mn^2+^-doped Cs_4_PbX_6_ perovskite were unstable upon exposure to high-energy electron beam and tend to damage within short period of time. XPS was performed on the powder samples using VG Multilab 2000 instruments to identify the chemical state of the elements on the sample surface (below 10 nm), employing Al as the target anode for the X-ray generation. The energy of the X-ray photon (Al Kα) is 1480 eV with a line width of 0.5 eV. The chemical compositions of the synthesized samples were analyzed using ICP-OES (Perkin-Elmer, OPTIMA 8300), at KBSI, Gwangju, South Korea. ESR measurement was carried out using JEOL JES-FA200 with an X-band microwave frequency of 9.17 GHz, with sample dispersion in n-octane. The liquid samples were taken in an ESR tube and frozen in liquid nitrogen at 173 K. All the spectra were recorded using the following ESR parameters: microwave power, 1 mW; modulation amplitude, 0.2 mT; modulation frequency, 100 kHz; and sweep time, 120 s.

Photoluminescence was measured using a Hitachi F-4500 fluorescence spectrophotometer over the wavelength range of 200 to 750 nm, using n-octane dispersions in a 1×1 cm quartz cuvette. UV-vis spectra were obtained using Optizen POP UV/Vis spectrophotometer. The lifetime was measured using a time-correlated single photon counting (TCSPC) system on FL920 Edinburgh instruments at Korean Advance Institute of Science and Technology (KAIST), South Korea. Pulsed laser irradiation with 300 nm was used as the excitation source. Internal quantum efficiency was measured with 365 and 290 nm excitation using a xenon laser (Hamamatsu C9920-02) at the Korea Photonics Technology Institute (KOPTI), South Korea. Time-resolved photoluminescence measurement for Mn^2+^ (600 nm) and D-state emission (420 nm for Cs_4_Pb(Br/Cl)_6_ and 400 nm for Cs_4_PbCl_6_) was carried out using single-mode pulsed diode laser as the excitation source (*λ*_max_ = 375 nm) at Korea Basic Science Institute (KBSI), Daegu Center, South Korea.

## Electronic supplementary material

Supplementary Information

## Data Availability

The data that support the findings of this study are available from the corresponding author upon request.

## References

[1] Protesescu L (2015). Nanocrystals of cesium lead halide perovskites (CsPbX_3_, X= Cl, Br, and I): novel optoelectronic materials showing bright emission with wide color gamut. Nano Lett..

[2] Huang H, Bodnarchuk MI, Kershaw SV, Kovalenko MV, Rogach AL (2017). Lead halide perovskite nanocrystals in the research spotlight: stability and defect tolerance. ACS Energy Lett..

[3] Saidaminov MI (2016). Pure Cs_4_PbBr_6_: highly luminescent zero-dimensional perovskite solids. ACS Energy Lett..

[4] Yang X (2018). Efficient green light-emitting diodes based on quasi-two-dimensional composition and phase engineered perovskite with surface passivation. Nat. Commun..

[5] Saidaminov MI, Mohammed OF, Bakr OM (2017). Low-dimensional-networked metal halide perovskites: the next big thing. ACS Energy Lett..

[6] Bakr OM, Mohammed OF (2017). Powering up perovskite photoresponse. Science.

[7] Ahmed GH (2017). Pyridine-induced dimensionality change in hybrid perovskite nanocrystals. Chem. Mater..

[8] Akkerman QA, Meggiolaro D, Dang Z, De Angelis F, Manna L (2017). Fluorescent alloy CsPb_x_Mn_1–x_I_3_ perovskite nanocrystals with high structural and optical stability. ACS Energy Lett..

[9] Meinardi F (2017). Doped halide perovskite nanocrystals for reabsorption-free luminescent solar concentrators. ACS Energy Lett..

[10] Yin J (2017). Molecular behavior of zero-dimensional perovskites. Sci. Adv..

[11] Andrews, R. H., Clark, S. J., Donaldson, J. D., Dewan, J. C., & Silver, J. Solid-state properties of materials of the type Cs_4_MX_6_ (where M=Sn or Pb and X=Cl or Br). *J. Chem. Soc. Dalton Trans.* **0**, 767–770 (1983).

[12] Nikl M, Mihokova E, Nitsch K (1992). Photoluminescence & decay kinetics of Cs_4_PbCl_6_ single crystals. Solid State Commun..

[13] Nikl M (1999). Photoluminescence of Cs_4_PbBr_6_ crystals and thin films. Chem. Phys. Lett..

[14] Kondo S (2001). Fundamental optical absorption of Cs_4_PbCl_6_. Solid State Commun..

[15] Kondo S, Amaya K, Saito T (2002). Localized optical absorption in Cs_4_PbBr_6_. J. Phys. Condens. Matter.

[16] Chen D, Wan Z, Chen X, Yuan Y, Zhong J (2016). Large-scale room-temperature synthesis and optical properties of perovskite-related Cs_4_PbBr_6_ fluorophores. J. Mater. Chem. C..

[17] Li X (2017). All inorganic halide perovskites nanosystem: synthesis, structural features, optical properties and optoelectronic applications. Small.

[18] Akkerman QA (2017). Nearly monodisperse insulator Cs_4_PbX_6_ (X= Cl, Br, I) nanocrystals, their mixed halide compositions, and their transformation into CsPbX_3_ nanocrystals. Nano Lett..

[19] Yin J (2017). Intrinsic lead ion emissions in zero-dimensional Cs_4_PbBr_6_ nanocrystals. ACS Energy Lett..

[20] Zhang Y (2017). Zero-dimensional Cs_4_PbBr_6_ perovskite nanocrystals. J. Phys. Chem. Lett..

[21] De Bastiani M (2017). Inside perovskites: quantum luminescence from bulk Cs_4_PbBr_6_ single crystals. Chem. Mater..

[22] Mir WJ, Jagadeeswararao M, Das S, Nag A (2017). Colloidal Mn-doped cesium lead halide perovskite nanoplatelets. ACS Energy Lett..

[23] Arunkumar P (2017). Colloidal organolead halide perovskite with a high Mn solubility limit: a step toward Pb-free luminescent quantum dots. J. Phys. Chem. Lett..

[24] Velázquez M (2008). Growth and characterization of pure and Pr^3+^-doped Cs_4_PbBr_6_ crystals. J. Cryst. Growth.

[25] Moller CK (1958). Crystal structure and photoconductivity of caesium plumbohalides. Nature.

[26] Luo YR, Kerr J (2012). Bond dissociation energies. CRC Handb. Chem. Phys..

[27] Zhang X (2017). Hybrid perovskite light-emitting diodes based on perovskite nanocrystals with organic–inorganic mixed cations. Adv. Mater..

[28] Lindblad R (2015). Electronic structure of CH_3_NH_3_PbX_3_ perovskites: dependence on the halide moiety. J. Phys. Chem. C..

[29] Palazon F (2017). Changing the dimensionality of cesium lead bromide nanocrystals by reversible postsynthesis transformations with amines. Chem. Mater..

[30] Liu Z (2017). Ligand mediated transformation of cesium lead bromide perovskite nanocrystals to lead depleted Cs_4_PbBr_6_ nanocrystals. J. Am. Chem. Soc..

[31] Swarnkar A, Mir WJ, Nag A (2018). Can B-site doping or alloying improve thermal- and phase-stability of all-inorganic CsPbX_3_ (X=Cl, Br, I) perovskites?. ACS Energy Lett..

[32] Nitsch K (1993). Growth and characterization of crystals of incongruently melting ternary alkali lead chlorides. Phys. Status Solidi A.

[33] Schlenker, C., Dumas, J., Greenblatt, M., & van Smaalen, S. *Physics and Chemistry of Low-Dimensional Inorganic Conductors* (Plenum press, New York, NY, 1996).

[34] Newman JA (2015). Parts per million powder X-ray diffraction. Anal. Chem..

[35] Folkerts HF, Ghianni F, Blasse G (1996). Search for D-level emission of Pb^2+^ in alkaline-earth aluminates and gallates. J. Phys. Chem. Solids.

[36] Das Adhikari S, Dutta SK, Dutta A, Guria AK, Pradhan N (2017). Chemically tailoring the dopant emission in manganese-doped CsPbCl_3_ perovskite nanocrystals. Angew. Chem. Int. Ed..

[37] Chen W (2001). Crystal field, phonon coupling and emission shift of Mn^2+^ in ZnS:Mn nanoparticles. J. Appl. Phys..

[38] Parobek D (2016). Exciton-to-dopant energy transfer in Mn-doped cesium lead halide perovskite nanocrystals. Nano Lett..

[39] Ishibashi A, Watanabe M, Hayashi T (1993). Evolution of excitonic states and localized exciton luminescence in Pb_1__–__*x*_Cd_*x*_I_2_ solid solutions. J. Phys. Soc. Jpn.

[40] Wang P (2018). Synthesis and characterization of Mn-doped CsPb(Cl/Br)_3_ perovskite nanocrystals with controllable dual-color emission. RSC Adv..

[41] Li F (2018). High Br–content CsPb(Cl_y_Br_1–y_)_3_ perovskite nanocrystals with strong Mn^2+^ emission through diverse cation/anion exchange engineering. ACS Appl. Mater. Interfaces.

[42] Kim YH (2017). A zero-thermal-quenching phosphor. Nat. Mater..

[43] Dirin DN (2016). Harnessing defect-tolerance at the nanoscale: highly luminescent lead halide perovskite nanocrystals in mesoporous silica matrixes. Nano Lett..

[44] Cho H (2015). Overcoming the electroluminescence efficiency limitations of perovskite light-emitting diodes. Science.

[45] Deschler F (2014). High photoluminescence efficiency and optically pumped lasing in solution-processed mixed halide perovskite semiconductors. J. Phys. Chem. Lett..

[46] Liu H (2017). CsPb_x_Mn_1–x_Cl_3_ perovskite quantum dots with high mn substitution ratio. ACS Nano.

[47] Yunakova O, Miloslavsky V, Kovalenko E, Kovalenko V (2015). Exciton absorption spectrum of Cs_4_PbCl_6_thin films. Funct. Mater..

[48] Yettapu GR (2016). Terahertz conductivity within colloidal CsPbBr_3_ perovskite nanocrystals: remarkably high carrier mobilities and large diffusion lengths. Nano Lett..

[49] Li J (2016). Temperature-dependent photoluminescence of inorganic perovskite nanocrystal films. RSC Adv..

[50] Zhang Q (2016). High-quality whispering-gallery-mode lasing from cesium lead halide perovskite nanoplatelets. Adv. Funct. Mater..

[51] Momma K, Izumi F (2008). VESTA: a three-dimensional visualization system for electronic and structural analysis. J Appl Crystallogr.

