# Publisher Correction: Probing molecule-like isolated octahedra via phase stabilization of zero-dimensional cesium lead halide nanocrystals

**DOI:** 10.1038/s41467-018-07910-7

**Published:** 2018-12-17

**Authors:** Paulraj Arunkumar, Han Bin Cho, Kyeong Hun Gil, Sanjith Unithrattil, Yoon Hwa Kim, Won Bin Im

**Affiliations:** School of Materials Science and Engineering, Chonnam National University, 77, Yongbong-ro, Buk-Gu, Gwangju 61186 Republic of Korea

Correction to: *Nature Communications*; 10.1038/s41467-018-07097-x, published online 08 November 2018

The original version of this Article contained an error in the title, which incorrectly read ‘Probing molecule-like isolated octahedra via—phase stabilization of zero-dimensional cesium lead halide nanocrystals.’ The correct version states ‘via phase stabilization’ in place of ‘via—phase stabilization’. This has been corrected in both the PDF and HTML versions of the Article.

