# Supplementary material for: Probing molecule-like isolated octahedra via phase stabilization of zero-dimensional cesium lead halide nanocrystals
Source: Nat Commun. 2018 Nov 8;9:4691. doi: 10.1038/s41467-018-07097-x (PMC6224409; doi:10.1038/s41467-018-07097-x)
Supplement: Supplementary file 1 — Supplementary Information [file 41467_2018_7097_MOESM1_ESM.docx]

Supplementary Information

Probing molecule-like isolated octahedra – Phase stabilization of zero-dimensional cesium lead halide nanocrystals

Paulraj Arunkumar^†^, Han Bin Cho^†^, Kyeong Hun Gil, Sanjith Unithrattil, Yoon Hwa Kim, and Won Bin Im^*^

*School of Materials Science and Engineering, Chonnam National University, 77, Yongbong-ro, Buk-Gu, Gwangju 61186, Republic of Korea*

**Supplementary Figures**

**
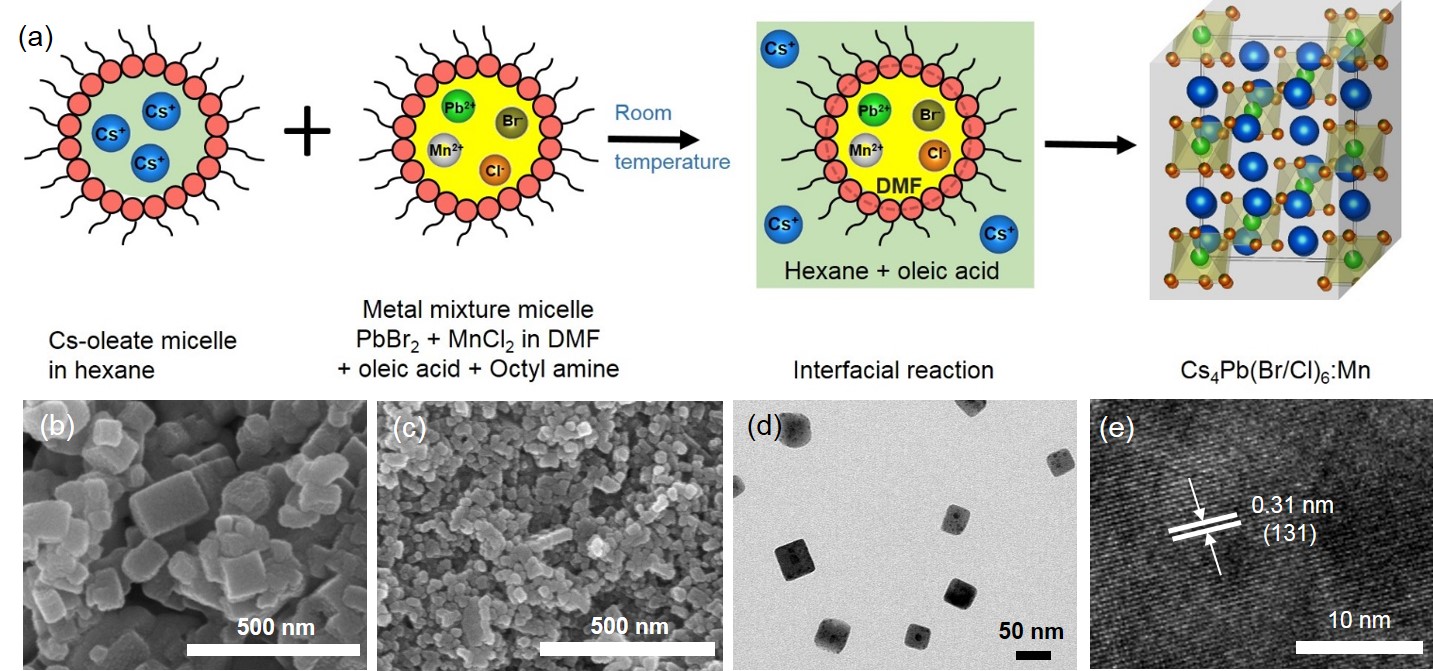
**

**Supplementary Figure 1.** Reverse microemulsion scheme employed for the synthesis of Mn^2+^-doped Cs_4_Pb(Br/Cl)_6_.


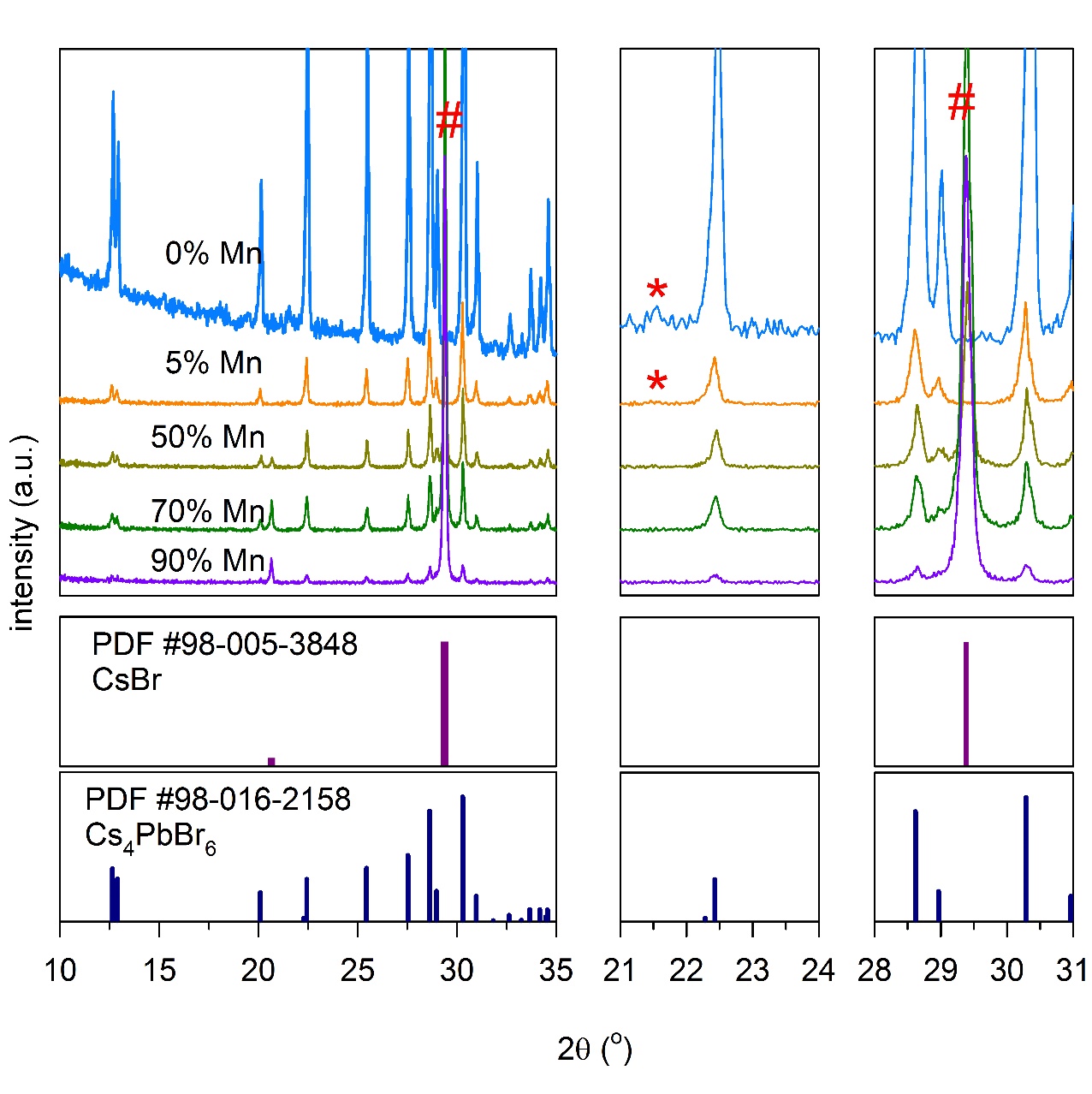


**Supplementary Figure 2.** XRD of undoped and Mn-doped Cs_4_PbBr_6_. The magnified Bragg’s angle in the range of 21 to 24^o^ and 28 to 31^o^ depicts the presence of secondary 3D CsPbBr_3_ phase denoted by asterix (*) and CsBr formation denoted by #, respectively. The secondary 3D CsPbBr_3_ phase were observed for 0 to 5% Mn, while the segregation of CsBr at higher Mn concentration above 50%.


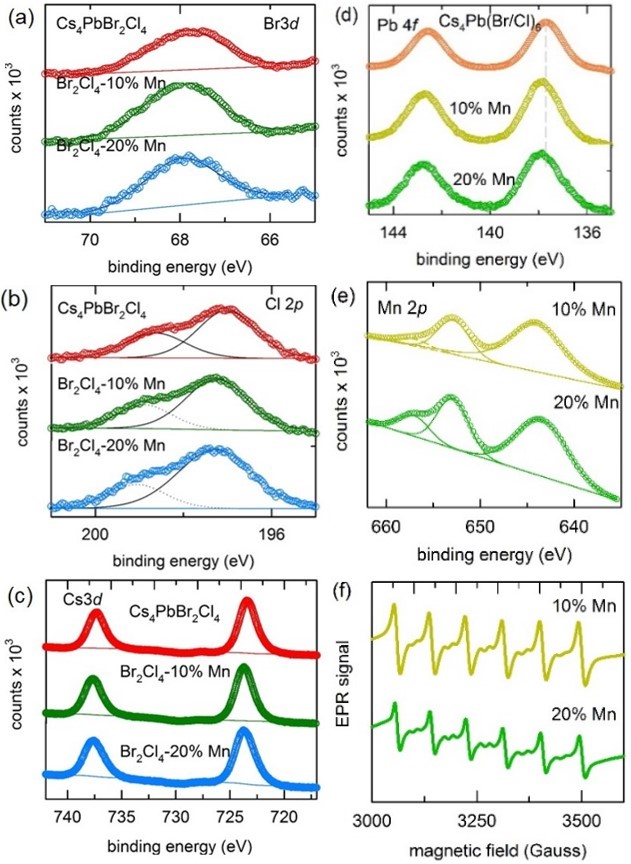


**Supplementary Figure 3.** XPS of (a) Br 3*d*, (b) Cl 2*p*, (c) Cs 3*d*, (d) Pb 4*f*, (e) Mn 2*p* core levels for the Cs_4_Pb(Br/Cl)_6_ sample with 10% and 20% Mn content. (f) low temperature ESR spectra of Cs_4_Pb(Br/Cl)_6_ colloids with varying Mn contents measured at 173 K.


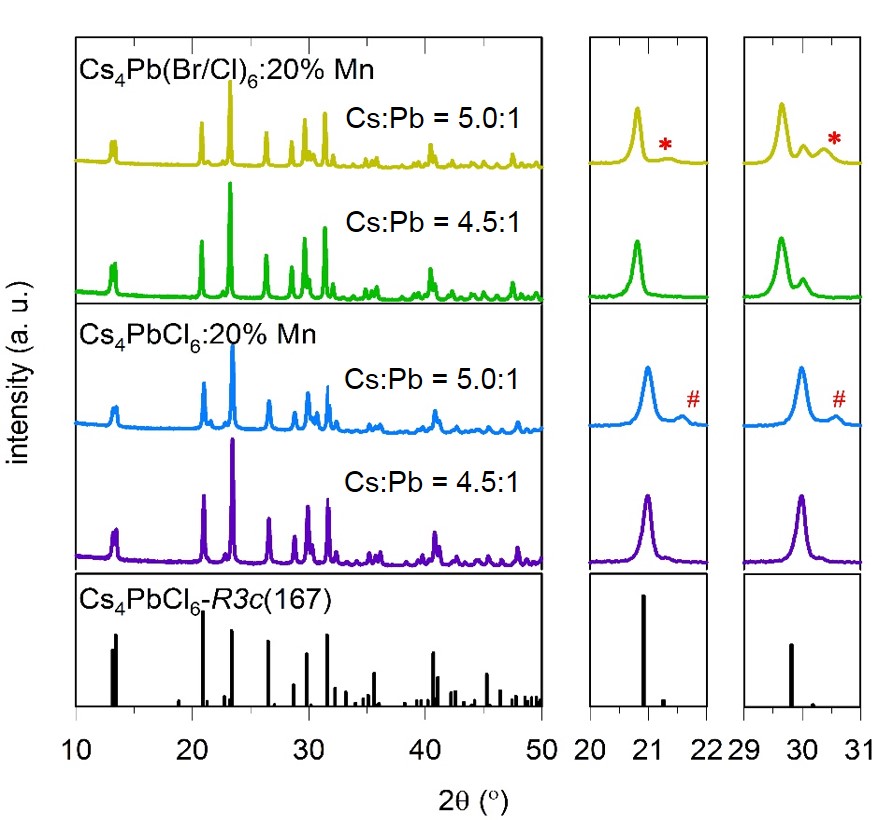


**Supplementary Figure 4.** XRD of CsPbX_6_ with 20% Mn doping with a reduced Cs:Pb mole ratio (4.5:1) *via* decreasing the Cs-oleate precursor, than the usual Cs:Pb mole ratio of (5.0:1). The magnified Bragg’s angle in the range of 20 to 22^o^ and 29 to 31^o^ indicates the formation of secondary Cs(Br/Cl) and CsCl phases, denoted by the ***** and **#**, respectively.


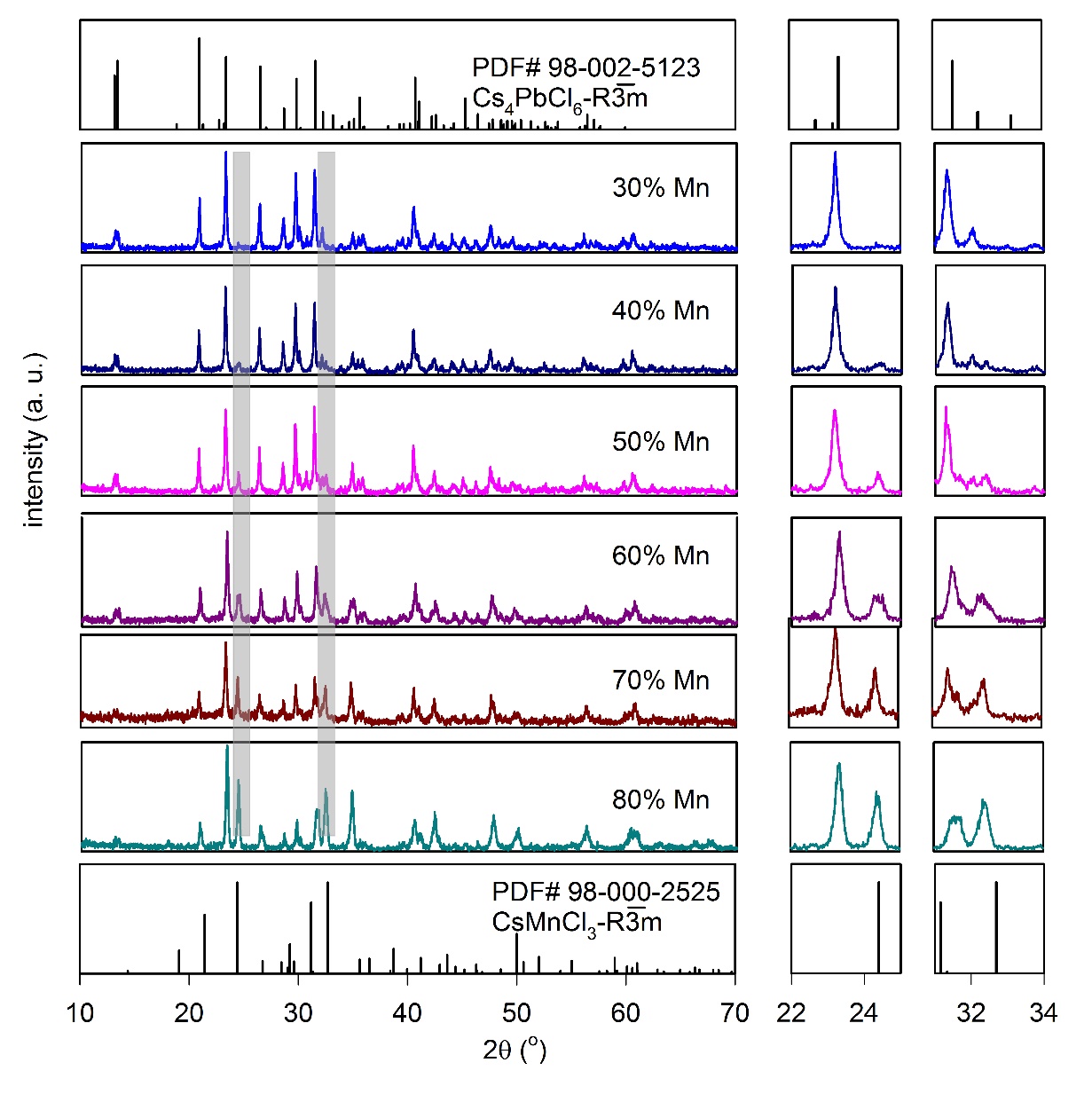


**Supplementary Figure 5.** XRD result of Mn-doped Cs_4_Pb(Br/Cl)_6_ with increasing Mn concentration upto 80% Mn. The magnified Bragg’s angle in the range of 22 to 25^o^ and 31 to 34^o^ indicates the formation of secondary CsMnCl_3_ phase (denoted by the shaded bars) coexisting with the 0D Cs_4_Pb(Br/Cl)_6_ phase with increasing Mn concentration, significantly affecting the optical properties of the perovskites. The undoped Cs_4_Pb(Br/Cl)_6_ composition is Cs_4_PbBr_2_Cl_4_.


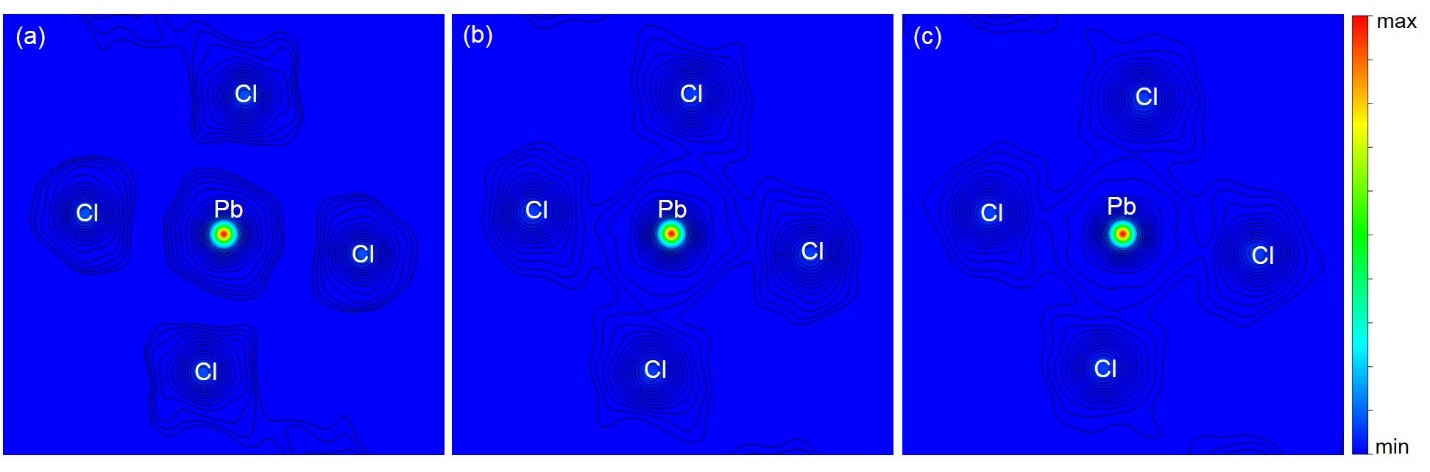


**Supplementary Figure 6.** Electron density distribution contour profile from X-ray diffraction derived Maximum Entropy method for the (a) undoped Cs_4_PbCl_6_, (b) Cs_4_PbCl_6_:10% Mn, and (c) Cs_4_PbCl_6_:20% Mn.


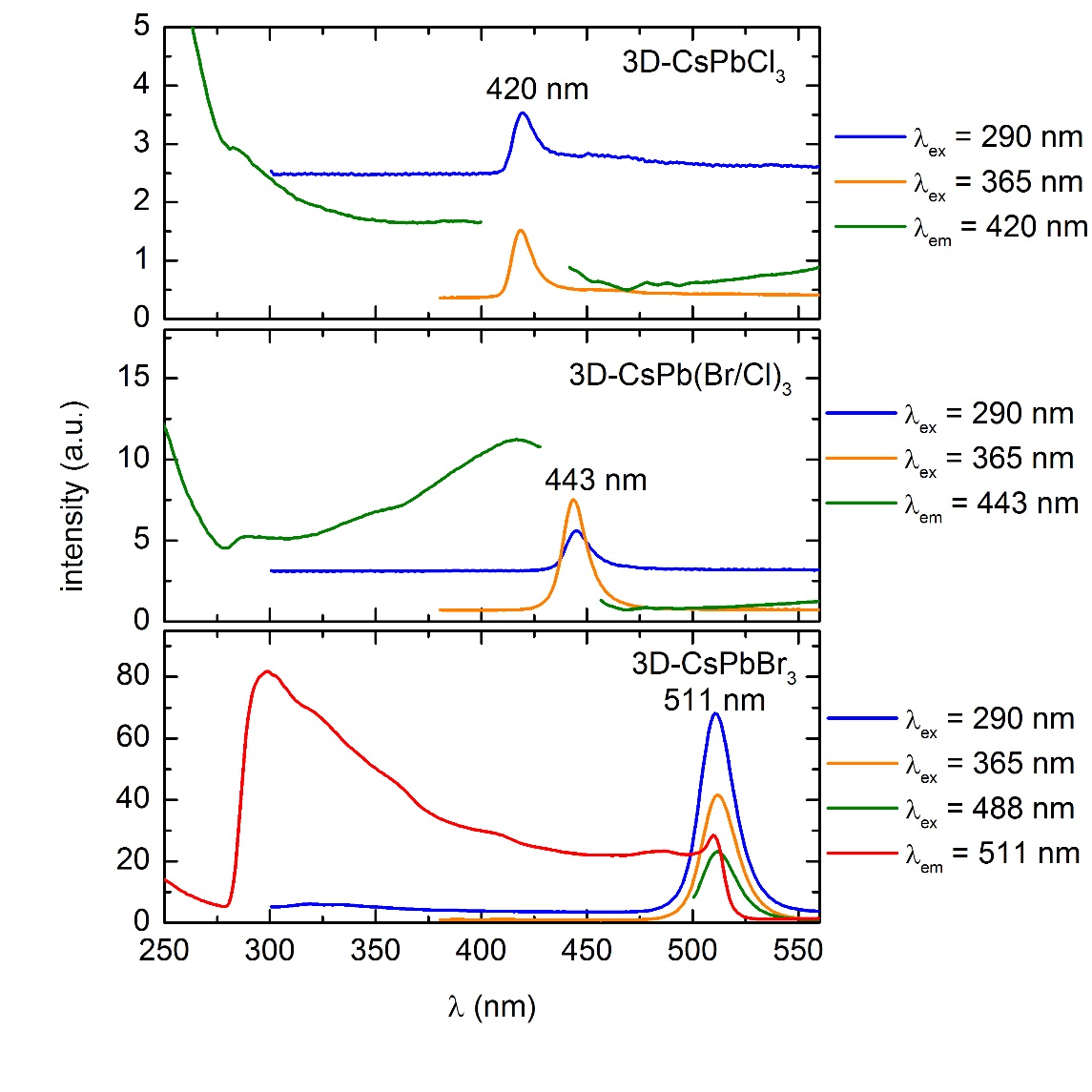


**Supplementary Figure 7.** PL and PLE spectra of pure 3D CsPbX_3_ nanocrystals where X = (a) Br, (b) Br/Cl, and (C) Cl, synthesized by hot-injection method.[^1^](#_ENREF_1)

**
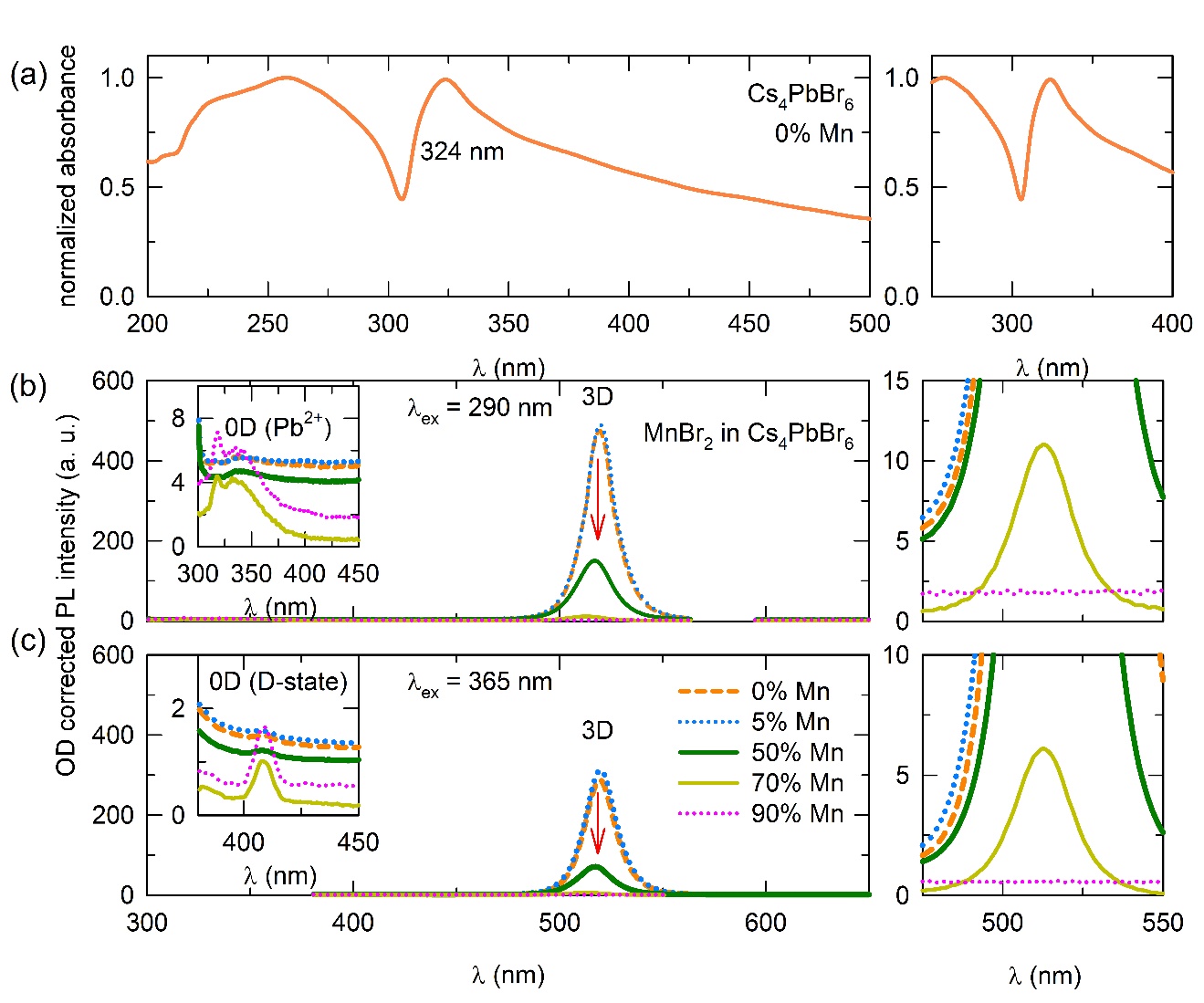
**

**Supplementary Figure 8.** (a) UV-Vis spectra of undoped Cs_4_PbBr_6_, and PL spectra of Mn^2+^-doped Cs_4_PbBr_6_ nanocrystals excited under (b) 290 nm and (c) 365 nm. The inset contains the magnified spectra of (b) Pb^2+^ and (c) D-state emissions. The green emission at 512 nm corresponding to the 3D CsPbBr_3_ impurity which decreases with increasing the Mn content. The sharp peak at 410 nm assigned to the octane solvent (70% and 90% Mn) which arises when emission intensity of samples becomes too low. The above results further demonstrate the phase stabilization of zero-dimensional Cs_4_PbX_6_ upon Mn incorporation for X = Br, including the Cl, and Br/Cl.


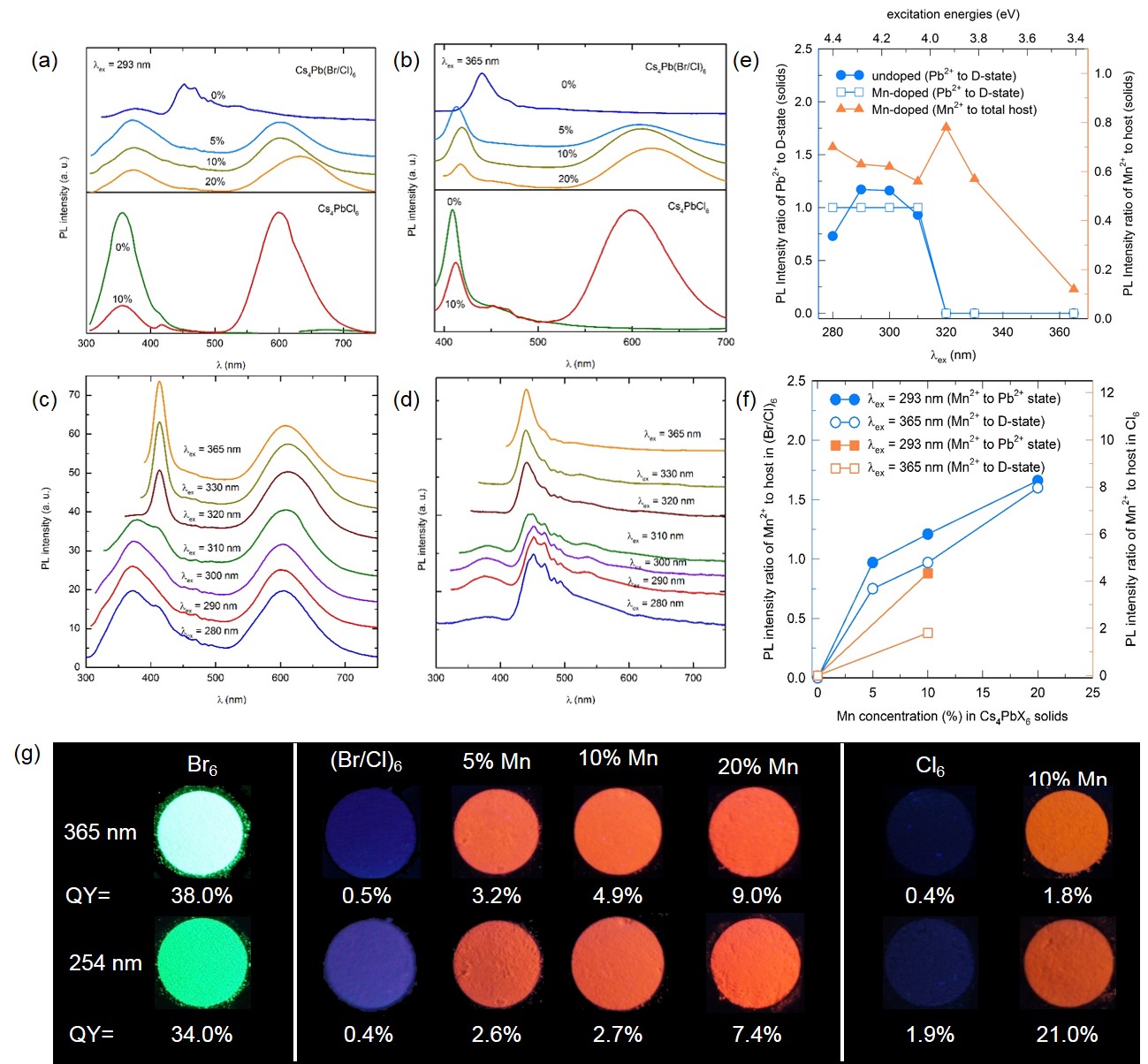


**Supplementary Figure 9.** PL spectra of Cs_4_PbX_6_ solids with varying Mn concentration by exciting at (a) 293 nm and (b) 365 nm. The variable excitation wavelengths from 280 to 365 nm were used for (c) 5% Mn-doped and (d) undoped Cs_4_Pb(Br/Cl)_6_, respectively_._ The PL intensity ratio of (e) Pb^2+^ to D-state and Mn^2+^ to the total host for the Cs_4_Pb(Br/Cl)_6_:5% Mn solids. (f) Mn concentration dependent PL intensity ratio of Mn^2+^ to the host states in Cs_4_PbX_6_ solids where X = (Br/Cl) and Cl.


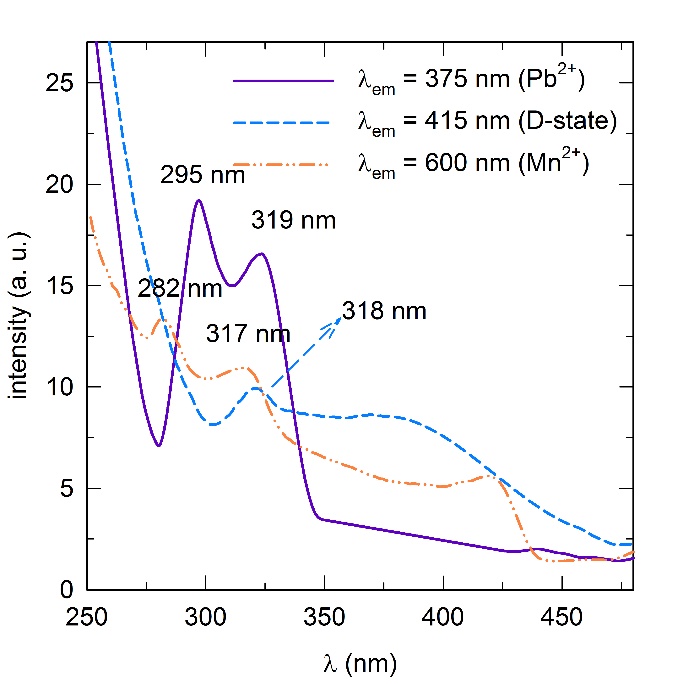


**Supplementary Figure 10.** Excitation spectrum of Cs_4_Pb(Br/Cl)_6_:5% Mn solids monitored at 375 nm (Pb^2+^ state), 415 nm (D-states), and 600 nm (Mn^2+^) emission.

**
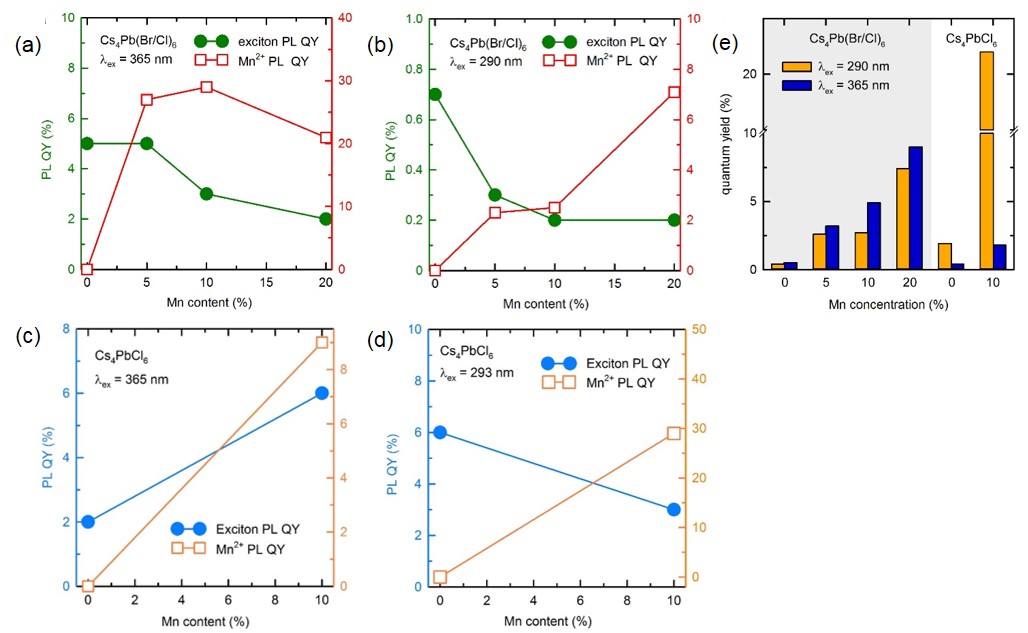
**

**Supplementary Figure 11.** PL QY of (a, b) Cs_4_Pb(Br/Cl)_6_ and (c, d) Cs_4_PbCl_6_ colloids for exciton and Mn^2+^ emission with varying Mn content under (a, c) 365 and (b, d) 293 nm excitation, respectively. (e) PL quantum yield of Cs_4_Pb(Br/Cl)_6_ and Cs_4_PbCl_6_ solids (powder) under 290 and 365 nm excitation.


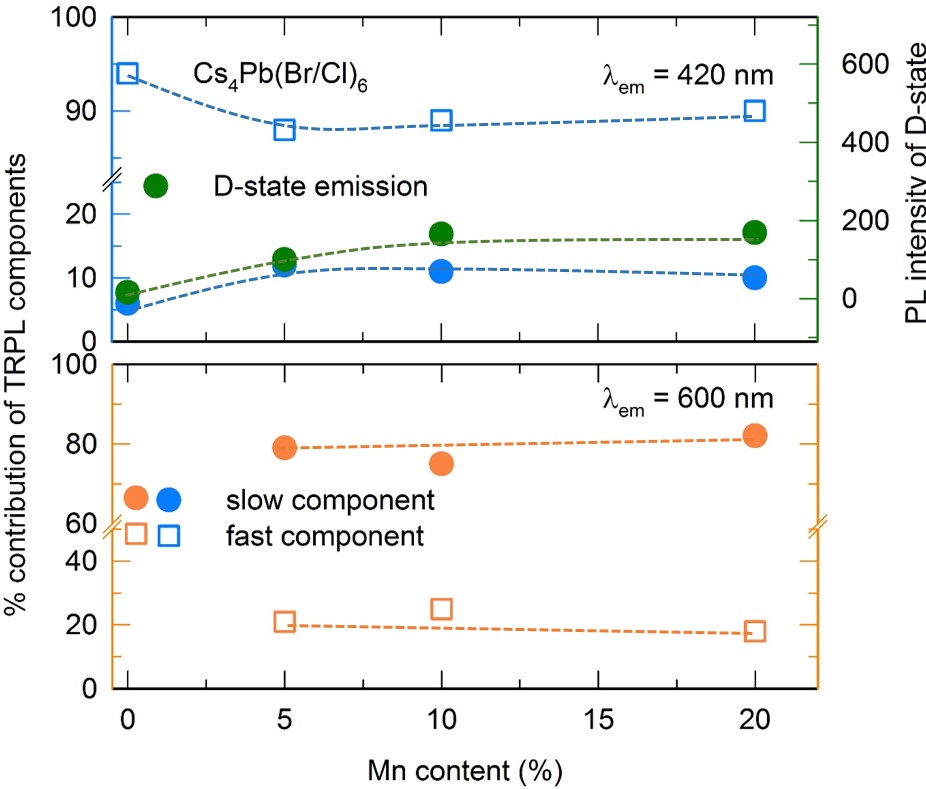


**Supplementary Figure 12.** Time resolved PL (TRPL) contribution of decay components in Cs_4_Pb(Br/Cl)_6_ colloids monitoring the emission at 420 and 600 nm describing the radiative (slow component, τ_1_) and non-radiative (fast components, τ_2+_ τ_3_). The optical density corrected PL intensity of D-state (as shown in Fig. 2a) of Cs_4_Pb(Br/Cl)_6_ is employed for the comparison. The excitation wavelength employed here is 375 nm.

**
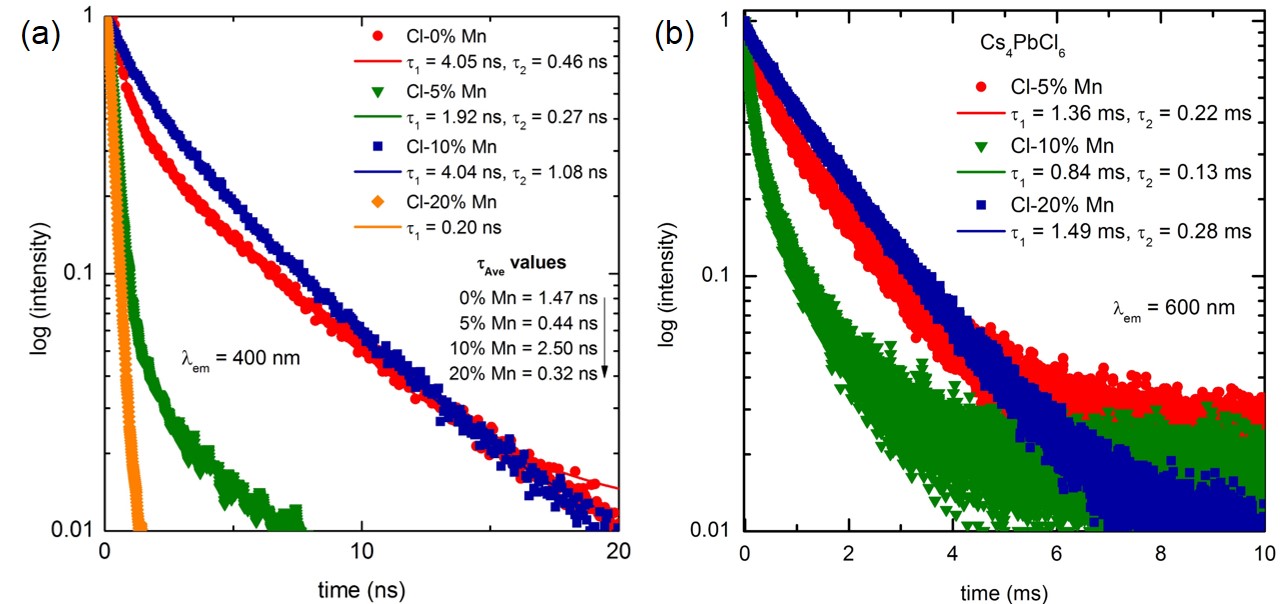
**

**Supplementary Figure 13.** PL decay of Cs_4_PbCl_6_ colloids with varying Mn content under 375 nm excitation and monitoring the emission at (a) 400 nm and (b) 600 nm.

**
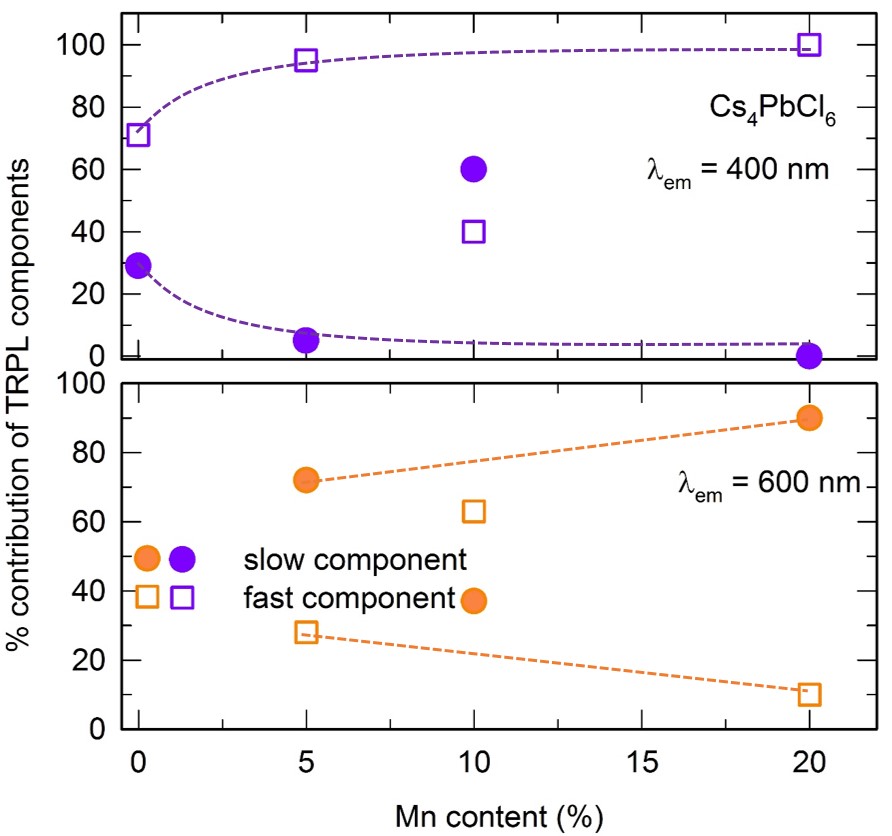
**

**Supplementary Figure 14.** TRPL contribution of decay components in Cs_4_PbCl_6_ colloids monitoring the emission at 400 and 600 nm describing the radiative (slow component, τ_1_) and non-radiative (fast components, τ_2_).

**
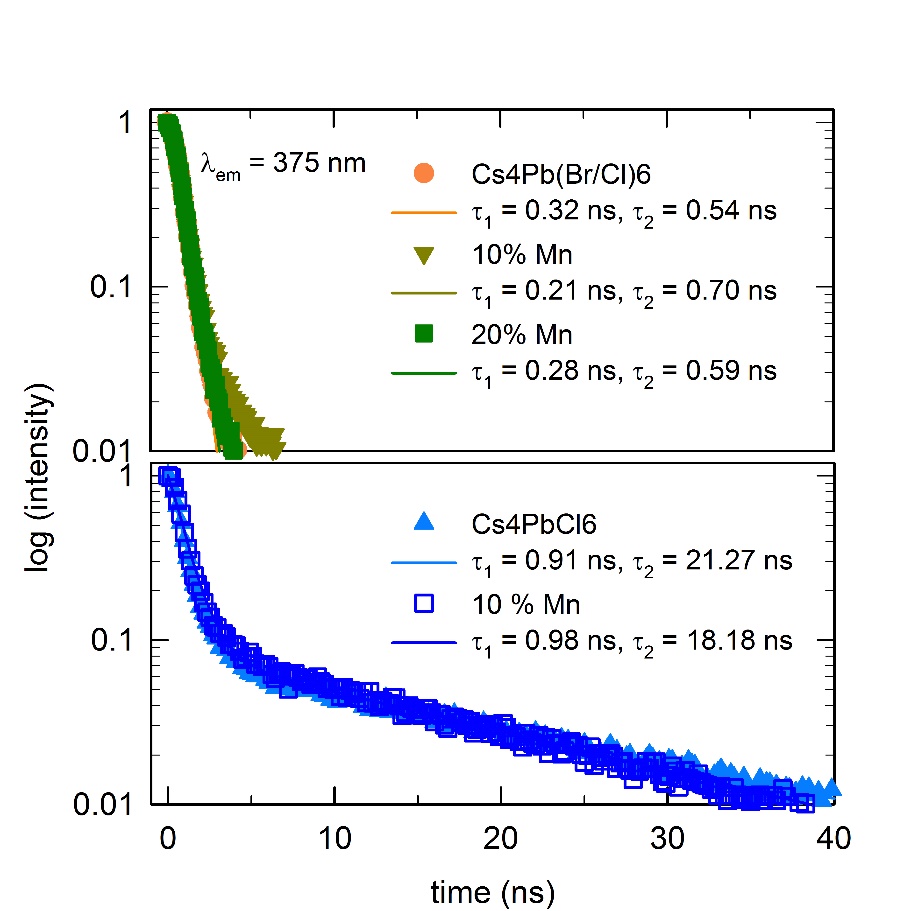
**

**Supplementary Figure 15.** PL decay of Cs_4_Pb(Br/Cl)_6_ and Cs_4_PbCl_6_ colloids with varying Mn content monitoring the 375 nm (Pb^2+^) emission, under 300 nm excitation.

**Supplementary Table 1.** Inductively coupled plasma-optical emission spectroscopy (ICP-OES) results of synthesized of Mn^2+^-doped Cs_4_Pb(Br/Cl)_6_ nanocrystals. The composition of undoped Cs_4_Pb(Br/Cl)_6_ is (Cs_4_PbBr_2_Cl_4_).

| target composition | target  (atomic %) | | observed  (atomic %) | | | observed composition |
| --- | --- | --- | --- | --- | --- | --- |
| *x*/Mn | Pb | Mn | | Pb | Mn | *x*/Mn |
| Cs_4_Pb(Br/Cl)_6_ | 100 | 0 | | 100 | 0 | 0 |
| Cs_4_Pb(Br/Cl)_6_ :10% Mn | 90 | 10 | | 90 | 10 | 0.10 |
| Cs_4_Pb(Br/Cl)_6_ :20% Mn | 80 | 20 | | 84 | 16 | 0.16 |

**Supplementary Table 2.** Structural parameters of Cs_4_PbBr_6,_ undoped and doped Cs_4_PbCl_6_, calculated using the Rietveld refinement. The number in the parentheses are the estimated standard deviation of the last significant figure.

| formula | Cs_4_PbBr_6_ | Cs_4_PbCl_6_ | Cs_4_PbCl_6_:10% Mn |
| --- | --- | --- | --- |
| space group | *R*$\bar{3}$*c* | *R*$\bar{3}$*c* | *R*$\bar{3}$*c* |
| composition | Cs_4_PbBr_6_ | Cs_4_PbCl_6_ | Cs_4_Pb_0.9_Mn_0.1_Cl_6_ |
| Wt. fraction | 95% | 96% | 97% |
| *a*/Å | 13.7188(1) | 13.1825(1) | 13.1370(1) |
| *c*/Å | 17.2931(3) | 16.6140(3) | 16.6169(2) |
| *V*/Å^3^ | 2818.61(5) | 2500.34(5) | 2497.16(4) |
| *Z* | 6 | 6 | 6 |
| space group | *Pm*$\bar{3}$*m* | *Pm*$\bar{3}$*m* | *Pm*$\bar{3}$*m* |
| composition | CsBr | 3D CsPbCl_3_ | 3D CsPbCl_3_ |
| Wt. fraction | 5% | 4% | 3% |
| *a*/Å | 4.2960 | 5.5990 | 5.5990 |
| *V*/Å^3^ | 79.29 | 175.52 | 175.52 |
| *Z* | 1 | 1 | 1 |
| *Χ*^2^ | 2.89 | 2.77 | 2.83 |
| *R_wp_* (%) | 5.66 | 5.58 | 5.60 |
| *R_p_* (%) | 4.39 | 4.20 | 4.24 |

**Supplementary Table 3.** Rietveld refined parameters of Cs_4_Pb(Br/Cl)_6_ (Cs_4_PbBr_2_Cl_4_) QDs. The number in the parentheses are the estimated standard deviations of the last significant figure.

| primary phase : 0D Cs_4_PbBr_2_Cl_4_ | | | | | | |
| --- | --- | --- | --- | --- | --- | --- |
| atomic parameters | | | | | | |
| atom | site | *x* | *y* | *z* | occupancy | *U* [Å^2^] |
| Pb | 6*b* | 0 | 0 | 0 | 1.0 | 0.0140(3) |
| Cs1 | 6*a* | 0 | 0 | 1/4 | 1.0 | 0.0481(11) |
| Cs2 | 18*e* | 0.3729(18) | 0 | 1/4 | 1.0 | 0.0262(7) |
| Br | 36*f* | 0.1964(4) | 0.0299(23) | 0.1023 | 0.3386(2) | 0.0288(15) |
| Cl | 36*f* | 0.1964(4) | 0.0299(23) | 0.1023 | 0.6714(2) | 0.0288(15) |
|  |  |  |  |  |  |  |
| secondary phase : 3D CsPbBr_3_ | | | | | | |
| atom | site | *x* | *y* | *z* | occupancy | *U*  [Å^2^] |
| Pb | 1*a* | 1.0 | 0 | 0 | 0 | 0.090(19) |
| Cs | 1*b* | 1.0 | 1/2 | 1/2 | 1/2 | 0.0854(16) |
| Br | 12*h* | 0.25 | 0.98741 | 1/2 | 0 | 0.0176(17) |

**Supplementary Table 4.** Rietveld refined parameters of Cs_4_Pb(Br/Cl)_6_:10% Mn (Cs_4_Pb_0.9_Mn_0.1_Br_1.8_Cl_4.2_). The number in the parentheses are the estimated standard deviations of the last significant figure.

| primary phase : 0D Cs_4_Pb(Br/Cl)_6_:10% Mn | | | | | | | | | | | | |
| --- | --- | --- | --- | --- | --- | --- | --- | --- | --- | --- | --- | --- |
| atomic parameters | | | | | | | | | | | | |
| atom | site | | *x* | | *y* | | *z* | | | occupancy | *U* [Å^2^] | |
| Pb | 6*b* | | 0 | | 0 | | 0 | | | 0.9 | 0.0118(7) | |
| Mn | 6*b* | | 0 | | 0 | | 0 | | | 0.1 | 0.0118(7) | |
| Cs1 | 6*a* | | 0 | | 0 | | 1/4 | | | 1.0 | 0.0483(13) | |
| Cs2 | 18*e* | | 0.3727(13) | | 0 | | 1/4 | | | 1.0 | 0.0330(7) | |
| Br | 36*f* | | 0.1943(27) | | 0.0300(29) | | 0.1014(17) | | | 0.3 | 0.0377(13) | |
| Cl | 36*f* | | 0.1943(27) | | 0.0300(29) | | 0.1014(17) | | | 0.7 | 0.0377(13) | |
|  | |  | |  | |  | |  | |  | |  |
| secondary phase : 3D CsPbBr_3_ | | | | | | | | | | | | |
| atom | | site | | *x* | | *y* | | | *z* | occupancy | *U* [Å^2^] | |
| Pb | | 1*a* | | 0 | | 0 | | | 0 | 1 | 0.0339 | |
| Cs | | 1*b* | | 1/2 | | 1/2 | | | 1/2 | 1 | 0.0573 | |
| Br | | 12*h* | | 0.98692 | | 1/2 | | | 0 | 0.25 | 0.1418 | |

**Supplementary Table 5.** Atomic bond distances of the undoped and Mn-doped Cs_4_Pb(Br/Cl)_6_ with 5%, 10%, and 20% Mn. The numbers in the parenthesis indicated the standard deviation of the least significant figures.

| atoms |  | bond lengths (Å) | | | |  |
| --- | --- | --- | --- | --- | --- | --- |
|  | Cs_4_Pb(Br/Cl)_6_ | | 5% Mn | 10% Mn | 20% Mn | |
| Pb-(Br/Cl) × 6 | 2.9910(4) | | 2.972(4) | 2.9503(28) | 2.9280(5) | |
| Mn-(Br/Cl) × 6 | - | | 2.972(4) | 2.9503(28) | 2.9280(5) | |
| Cs1-(Br/Cl) × 6 | 3.4880(4) | | 3.4810(4) | 3.4671(31) | 3.4680(4) | |
| Cs2-(Br/Cl) × 2 | 3.5820(4) | | 3.5890(4) | 3.5748(34) | 3.5600(4) | |
| Cs2-(Br/Cl) × 2 | 3.5850(4) | | 3.5960(4) | 3.5802(31) | 3.5710(6) | |
| Cs2-(Br/Cl) × 2 | 3.5990(4) | | 3.6000(5) | 3.5993(31) | 3.6050(5) | |
| Cs2-(Br/Cl) × 2 | 3.7560(5) | | 3.7490(4) | 3.7450(4) | 3.7260(5) | |

**Supplementary Table 6.** Atomic bond distances of the Cs_4_PbBr_6_, undoped and Mn-doped Cs_4_PbCl_6_ with 10% Mn. The numbers in the parenthesis indicated the standard deviation of the least significant figures.

| atoms | bond lengths (Å) | atoms | bond lengths (Å) | |
| --- | --- | --- | --- | --- |
|  | Cs_4_PbBr_6_ |  | Cs_4_PbCl_6_ | Cs_4_PbCl_6_:10% Mn |
| Pb-Br × 6 | 3.0470(3) | Pb-Cl × 6 | 2.9330(7) | 2.9030(6) |
| Mn-Br × 6 | - | Mn-Cl × 6 | - | 2.9030(6) |
| Cs1-Br × 6 | 3.5830(3) | Cs1-Cl × 6 | 3.4380(7) | 3.4360(6) |
| Cs2-Br × 2 | 3.6760(3) | Cs2-Cl × 2 | 3.5430(8) | 3.5390(6) |
| Cs2-Br × 2 | 3.6950(3) | Cs2-Cl × 2 | 3.5460(7) | 3.5550(6) |
| Cs2-Br × 2 | 3.7280(3) | Cs2-Cl × 2 | 3.5730(7) | 3.5750(6) |
| Cs2-Br × 2 | 3.8370(3) | Cs2-Cl × 2 | 3.6900(7) | 3.6950(5) |

**Supplementary Table 7.** Surface compositions of the synthesized Mn-doped CsPb(Br/Cl)_6_ perovskites derived from X-ray photoelectron spectroscopy results.

| elemental composition | atomic. (%) | | | | |  |
| --- | --- | --- | --- | --- | --- | --- |
| samples | CsPb(Br/Cl)_6_ | | CsPb(Br/Cl)_6_-10%Mn | | CsPb(Br/Cl)_6_-20%Mn | |
| elements | expected | observed | expected | observed | expected | observed |
| Cs | 4 | 4 | 4 | 4 | 4 | 4 |
| Pb | 1 | 1.49 | 0.90 | 1.93 | 0.80 | 1.02 |
| Mn | 0 | 0 | 0.10 | 0.33 | 0.20 | 0.48 |
| Cl | 4 | 3.73 | 4.2 | 1.89 | 4.4 | 3.30 |
| Br | 2 | 1.02 | 1.8 | 1.18 | 1.6 | 0.61 |
| Pb/Mn ratio | 1 | 1 | 9.00 | 5.85 | 4.00 | 2.12 |
| Cl/Br ratio | 2.00 | 3.66 | 2.33 | 1.60 | 2.75 | 5.40 |
| Pb/X ratio | 0.16 | 0.36 | 0.15 | 0.74 | 0.13 | 0.26 |
| Pb/Cs ratio | 0.25 | 0.37 | 0.23 | 0.57 | 0.20 | 0.26 |

**Supplementary Table 8.** Comparison of state-of-the-art PL QY of Mn^2+^ emission in Mn-doped perovskite colloids reported in the literature.

| Cs_4_PbX_6_ host | λ_ex_ (nm) | Mn^2+^ content  (mole%) | Mn QY  (%) | synthesis temperature | references |
| --- | --- | --- | --- | --- | --- |
| CsPbCl_3_:Mn | 365 | 9.6 | 27% | 185^o^C | [^2^](#_ENREF_2)J. Am. Chem. Soc., **2016**, 138, 14954–14961 |
| CsPbCl_3_:Mn | 365 | 0.2 | 22-58% | 120-200^o^C | [^3^](#_ENREF_3)Nano Lett., **2016**, 16, 7376–7380 |
| CsPbCl_3_:Mn | 365 | 0.8 | 20% | RT | [*^4^*](#_ENREF_4)*ACS Energy Lett*. **2017**, 2, 537−543 |
| CsPbCl_3_:Mn | 365 | 46 | 54%  (overall QY) | RT | [^5^](#_ENREF_5)ACS Nano, 2017, 11, 2239–2247 |
| Cs_4_Pb(Br/Cl)_6_:5% Mn | 365 | 5 | 27% | RT | Our results |
| Cs_4_Pb(Br/Cl)_6_:10% Mn | 365 | 10 | 29% | RT |  |
| Cs_4_PbCl_6_:10% Mn | 290 | 10 | 29% | RT |  |
| Cs_4_PbCl_6_:10% Mn solids | 290 | 10 | 21%  (solids) | RT |  |

**Supplementary Table 9.** PL quantum yield of the Cs_4_PbX_6_ solids.

| sample | λ_ex_ (nm) | total QY (%) | QY (%) | |  |
| --- | --- | --- | --- | --- | --- |
|  |  |  | excitonic emission (%) | Mn^2+^ emission (%) | |
| Cs_4_Pb(Br/Cl)_6_ | 365 | 0.3 | 0.3 | 0 | |
| Cs_4_Pb(Br/Cl)_6_-10%Mn | 365 | 3.0 | 0.1 | 2.9 | |
| Cs_4_PbCl_6_ | 293 | 1.0 | 1.2 | 0 | |
| Cs_4_PbCl_6_-10% Mn | 293 | 21.0 | 0.2 | 20.8 | |
| Cs_4_PbCl_6_-10% Mn | 365 | 2.0 | 0.1 | 1.9 | |
| Cs_4_PbBr_6_ | 365 | 38.0 | 38 | 0 | |

**Supplementary Note 1.**

Highly crystalline Mn-doped Cs_4_PbX_6_ nanocrystals could be obtained within few minutes *via* interfacial reaction at room temperature, through reverse microemulsion approach (Supplementary Figure 1). Mn-doped Cs_4_Pb(Br/Cl)_6_ were synthesized using Cs-oleate, PbBr_2_, MnCl_2_ precursors along with HCl. The halide ratio in the mixed halide (Br/Cl) series were estimated indirectly from the lead concentration for bromine and remaining halides were assigned to chlorine, due to the limitation of low sensitivity of Inductively coupled plasma optical emission spectroscopic technique (ICP-OES) to these halides. This technique may be reasonable since PbBr_2_ is the only source for bromine, while chlorine precursors are the MnCl_2_ and HCl, where lead and Mn concentration are estimated from ICP-OES.

**Supplementary Note 2.**

**XRD of Mn-doped Cs_4_PbBr_6_ nanocrystals**.

The secondary 3D CsPbBr_3_ phase were apparent in the 0 to 5% Mn-doped samples, and the segregation of CsBr at higher Mn concentration above 50% Mn. Unlike Br/Cl and Cl analogues, the Cs_4_PbBr_6_ necessitates higher Mn^2+^ concentration to stabilize the zero-dimensional structure. The reason may be attributed to the lower substitution ratio of Mn^2+^ in the bromine-dominated Cs-Pb-Br system (CsPbBr_3_ host)[^6^](#_ENREF_6). This causes a lack of sufficient lead/manganese precursor for reacting with the Cs-salt in the final composition of Cs_4_Pb_1-x_Mn_x_Br_6_, thereby leading to the segregation of excess CsBr salt at higher Mn-doping content. This anticipation was further evidenced from XRD results (Supplementary Figure 2) were CsBr segregation occurs at higher Mn doping concentration, particularly above 50% Mn content. It is to be noted that in the Br/Cl mixed halide phases, lower substitution ratio of Mn^2+^ were not observed, due to the presence of chloride dominant phase with a halide composition of Br_2_Cl_4_, and also evidenced from the asobtained Pb/Mn ratio in the ICP-OES results.

**Supplementary Note 3.**

**X-ray Photoelectron spectroscopy and Electron spin resonance results.**

The valence state, nature of bonds, and change in bond lengths upon Mn^2+^ incorporation in the Cs_4_Pb(Br/Cl)_6_ perovskite was further investigated by XPS measurements. The binding energy values of 142.5 (Pb 4*f*_5/2_) and 137.7 eV (Pb 4*f*_7/2_) confirms the presence of divalent Pb ions, whereas other elements (Cs 3*d,* Br 3*d*, Cl 2*p*) were found to be monovalent (Supplementary Figures 3a to 3e). The presence of Mn was confirmed from the Mn 2*p* spectrum. A marked shift in the Pb 4*f* peaks to higher binding energies is an indication of increase in the partial cationic charge of Pb ions due to Mn incorporation, and formation of strong bonding nature between Pb(Mn) and halide ions (Pb-X).[^7^](#_ENREF_7)^,^[^8^](#_ENREF_8) Similar peak shifts to higher binding energies were observed for all the elements with increasing Mn content. The ratio of Pb to halide and Cs species estimated from XPS measurements suggests Pb enrichment at the surface (Supplementary Table 7**)**. Electron spin resonance (ESR) of Mn-doped Cs_4_Pb(Br/Cl)_6_ colloids show a sextet hyperfine splitting, confirming the presence of Mn^2+^ ions at the isolated octahedrally coordinated PbX_6_ sites (Supplementary Figure 3f). The sharp hyperfine splitting even at higher Mn doping levels (20% Mn) originated from the weak interaction between neighboring Mn moieties even at high Mn contents, due to their presence within the isolated PbX_6_ octahedral units of Cs_4_PbX_6_. This further confirms the uniform dispersion of Mn^2+^ and the well-defined doping environments in the Cs_4_PbX_6_.[^4^](#_ENREF_4)

**Supplementary Note 4.**

**XRD of Mn-doped Cs_4_Pb(Br/Cl)_6_ with higher Mn concentration**. With the increase in the Mn doping concentration in Cs_4_Pb(Br/Cl)_6_, secondary CsMnCl_3_ phase were formed at above 40% Mn which further grown with increasing Mn-doping (below 80% Mn) as shown in Supplementary Figure 5. Temperature-induced conversion of 0D Cs_4_PbX_6_ to 3D CsPbX_3_ upon heating can be ignored during drying of powders, as drying temperature was limited to 80^o^C.

**XRD of Mn-doped Cs_4_Pb(Br/Cl)_6_**

A high stoichiometric Cs:Pb ratio of 5:1 were employed to favor the formation of Cs_4_PbX_6_ phase (for the synthesis of undoped to 20 % Mn concentration) and simultaneously avoid the Cs-deficient CsPbX_3_ (Cs:Pb = 1:1) phase. Despite using high-Cs content (Cs:Pb = 5:1), formation of undesired CsPbX_3_ phase were observed in the undoped samples. In addition, solvent washing was ineffective in removing the 3D chloride analogue (CsPbCl_3_) in the mixed-halide Cs_4_Pb(Br/Cl)_6_ during synthesis, due to its poor solubility in the organic solvents including dimethyl sulfoxide, isopropanol, and acetonitrile.

**Supplementary Note 5.**

**PL of Mn-doped Cs_4_PbX_6_ (X = Br, Cl) colloids**

Undoped Cs_4_PbBr_6_ nanocrystals exhibit two UV emission band and intense green emission (512 nm) arising from Pb^2+^/D-state emissions and 3D CsPbBr_3_ impurity, respectively (Supplementary Figure 8). Despite Cs_4_PbBr_6_ being a wide band gap material (3.95 eV)[^9^](#_ENREF_9) compared to CsPbCl_3_ (*E*_g_ of 2.8 eV), the former does not exhibit Mn^2+^ emission (2.10 eV) unlike latter. Typically, the Mn^2+^ emission has not been realized in the bromine dominant Cs-Pb-X host irrespective of its wider band gap. This may be attributed to the several competing optical processes that includes strong band-edge emission and its electron-hole recombination, and inefficient back energy transfer from host to Mn^2+^ ion particularly in the smaller band gap host, leading to weak or negligible Mn^2+^ emission in the bromine-dominant host[^6^](#_ENREF_6). In addition, the necessity of critical amount of chloride ion is anticipated to influence the Mn^2+^ emission *via* the energy transfer from host to strong Mn-Cl bond.

**Discussion on Figure 2c**: An intense Mn^2+^ emission with increasing Mn content, indicates an efficient energy transfer from host to Mn^2+^ ion in the wide band gap Cs_4_PbCl_6_ (4.37 eV)[^9^](#_ENREF_9). The excited ^3^P_1_ state of Pb^2+^ experiences an intense crystal field[^10^](#_ENREF_10) from high electronegative chlorine, leading to redshift of Pb^2+^ emission which then overlaps with D-state emission. Unlike Cs_4_Pb(Br/Cl)_6_, the disappearance of D-state emission in the chloride analogue cannot be assigned to suppressed charge transfer band, due to attributes of crystal field effects of chlorine on Pb^2+^ emission that comes into the picture.

**Supplementary Note 6.**

**PL properties of Mn-doped Cs_4_Pb(Br/Cl)_6_ solids (powders).**

The optical features of the Cs_4_PbX_6_ solids (powders are referred as ‘solids’) are consistent to those of colloids that includes the Mn^2+^-induced suppression of D-state emission, enhanced Mn^2+^ PL intensity at high excitation energies, and the dominant energy transfer from Pb^2+^ to Mn^2+^ rather than D-state.

The PL properties of Mn-doped Cs_4_PbX_6_ powders were investigated under 293 and 365 nm excitation (Supplementary Figure 9a, 9b). The undoped Cs_4_Pb(Br/Cl)_6_ solids exhibited emission at 450 nm (Supplementary Figure 9a) compared to colloids at 432 nm (Figure 2a) which is due to the quantum size effect of the 3D CsPb(Br/Cl)_3_ impurity phase where solids exist in their aggregated forms. A similar size dependent emission effect is also apparent under 365 nm excitation. While Pb^2+^ emission (372 nm) is largely independent of the particle size as deduced from the unchanged PL spectral positions. A shoulder at 533 nm for Cs_4_Pb(Br/Cl)_6_ is assigned to 3D CsPbBr_3_ impurity as supported by XRD results (Figure 1a). However, in the Mn-doped perovskite, the emission peak positions of solids are consistent with their colloidal form suggesting the presence of Mn^2+^ maintains the integrity of the nanocrystals and stabilization of the 0D perovskite, further utilizing the structural dimension effect of the perovskite. With Mn doping in Cs_4_Pb(Br/Cl)_6_, D-state band almost vanished completely, leaving out an intense Pb^2+^ (370 nm) emission that remains unchanged with increasing Mn concentration as shown in Supplementary Figure 9c, and is consistent with the colloidal samples. This reaffirmed that Mn^2+^ suppress the D-state band at higher excitation energies (>4.0 eV), by blocking the charge-transfer process in the isolated octahedrons. In the undoped Cs_4_Pb(Br/Cl)_6,_ the D-band emission at 450 nm remains unchanged in the excitation wavelength range of 280 to 365 nm, along with the additional Pb^2+^ emission (Supplementary Figure 9d). This demonstrates that in addition to facilitating D-band emission in the absence of Mn^2+^, an energy transfer from Pb^2+^ state to the D-band emission is evident, at the excitation wavelengths where only Pb^2+^ is excited (excitation wavelength range of 290 to 310 nm) as shown in Supplementary Figure 9d. In addition, the Mn^2+^ suppress the D-band emission particularly at higher excitation energies (≤310 nm), where Pb^2+^ is excited, suggesting the energy transfer from Pb^2+^ is efficient absorbed by Mn^2+^ over the D-states (Supplementary Figure 9e). A minor red shift of the Pb^2+^ (370 nm) emission was apparent for the Mn-doped sample, in the excitation wavelengths range (280 → 310 nm); this can be ascribed to the filling of lower-lying energy states (relative to the ^3^P_1_ levels) at the lower excitation energies. The enhanced PL intensity ratio of Mn^2+^ to Pb^2+^ with Mn concentration and red-shifted Mn^2+^ emission at 20% Mn solid sample is consistent with their colloidal form. Similarly, the Pb^2+^ (356 nm) and Mn^2+^ (600 nm) emissions were also retained in the Mn-doped Cs_4_PbCl_6_ when excited at 293 nm. The presence of 3D CsPbCl_3_ phase in the Cs_4_PbCl_6_:10% Mn solids were confirmed by the emission shoulder at ~420 nm. Intensity of Mn^2+^ emission is much larger for the wide band gap chloride analogues under 293 nm excitation due to its large absorption at high excitation energies (Supplementary Figure 9e-f). Therefore, the difference in D-state to Mn^2+^ orange emission ratio between mixed halide and its chloride analogue is controlled by the absorption strength at the employed excitation wavelength. The interesting fact is that unlike in colloids, the orange emission of Mn^2+^ is more predominant in the solid form due to an efficient energy transfer from exciton to Mn^2+^ and spatially confinement effect in the isolated octahedral units and localized excitons of 0D perovskites.

**Supplementary Note 7.**

**Excitation spectrum of Mn-doped Cs_4_Pb(Br/Cl)_6_ solids.** The energy transfer and direct excitation of the ^3^P_1_ levels of Pb^2+^ ion, D-state levels, and Mn^2+^ were further confirmed from their respective excitation spectrum (Supplementary Figure 10). A comparison of the excitation spectra for 375 (Pb^2+^ - UV emission), 415 (D-state), and 600 nm (Mn^2+^) suggest that in the excitation range of 290 to 310 nm, Mn^2+^ emission arise through the energy transfer from Pb^2+^ (UV emission), while at ≥320 nm excitation, Mn^2+^ emission arise due to the energy transfer from D-state. In addition, at 280 nm, a direct excitation of D-state is also clearly apparent, which competes well with the Mn^2+^ and Pb^2+^ states. Therefore, at 280 nm excitation, the Mn^2+^ emission arise from the combined excitation of Pb^2+^, D-state, and Mn^2+^ states. Fewer overlap of Mn^2+^ and D-state reveals a partial energy transfer from D-state to Mn^2+^ at >320 nm excitation. An energy transfer from Pb^2+^ states to D-states cannot be completely ruled out due to the increase in the D-state emission in the excitation wavelengths ranges of 290 to 310 nm, where the absorption of D-states is negligible, which also agrees with earlier reports.

**Supplementary Note 8.**

**PL QY of Mn-doped Cs_4_Pb(Br/Cl)_6_ colloids and powders.** Interestingly, the PL QY of the Cs_4_Pb(Br/Cl)_6_ colloids under 365 nm excitation retained the same value (QY = 5%) upon Mn doping, even after the energy transfer from host (D-state) to Mn^2+^ ions (Supplementary Figure 11a). This suggests that the presence of dopant states blocks energy leakages through trap states and enhances the host (D-state) emission, which is then involved in the energy transfer to Mn^2+^, ultimately resulting in the host retaining the same exciton PL QY.[^3^](#_ENREF_3) However, the enhanced energy transfer with increasing Mn concentration led to a highest Mn^2+^ PL QY of 29% for the Cs_4_Pb(Br_/_Cl)_6_:10% Mn (Supplementary Table 8); this represents the second highest value ever reported for Mn^2+^ emission in perovskite nanocrystals, next to the 54% value of Mn-doped 3D CsPbCl_3_.[^5^](#_ENREF_5) The efficient energy transfer to Mn^2+^, promoted by the localized excitons and the spatially confined 0D perovskite structure, is responsible for the maximum dopant QY of 29% in Cs_4_Pb(Br_/_Cl)_6_ perovskites. Furthermore, the effective utilization of high exciton binding energy of the Cs_4_PbX_6_ perovskites, achieved by blocking the energy leakage to traps in the presence of Mn^2+^, is responsible for the overall superior QY of the Cs_4_PbX_6_ host. The concentration quenching of Mn^2+^ luminescence decreased at 20% Mn, due to the stronger interaction between neighboring Mn ions in the nanocrystals.[^11^](#_ENREF_11) A decrease in the exciton PL QY with increasing Mn concentration in the Cs_4_Pb(Br/Cl)_6_ perovskite, further confirms the occurrence of an energy transfer to Mn^2+^. These results suggest that the high luminescent 0D perovskite nanocrystals doped with Mn^2+^ could represent promising emitting materials for designing efficient semiconductor-based solid-state lighting devices with minimum self-absorption. Additionally, the excitonic PL QY of Cs_4_PbCl_6_ increased from ~2 to 7% upon Mn doping under 365 nm excitation for the same reason of blocking of energy leakage through trap states by the dopant states (Supplementary Figure 11c).[^3^](#_ENREF_3) A low PL QY of 9% was obtained for Mn^2+^ emission in Cs_4_PbCl_6_, due to the lower absorption in the 365 nm range highlighted in the UV-vis spectra (Figure 2b).

In the case of the chloride analogue, PL QY of 29% was obtained for Cs_4_PbCl_6_:10% Mn, which is among the highest values for Mn^2+^ emission reported to date. This is due to the efficient energy transfer from Pb^2+^ to Mn^2+^ under high excitation energies of λ_ex_ = 290 nm (Supplementary Figure 11d). Therefore, high PL QY values for the Mn^2+^ emission were obtained for Cs_4_Pb(Br/Cl)_6_ and Cs_4_PbCl_6_ analogues at lower and higher excitation energies, respectively, due to their appropriate band gaps (as determined by the halide species), enabling efficient energy transfer to the Mn^2+^ levels. On the contrary, a low PL QY (<8%) of Cs_4_Pb(Br/Cl)_6_ perovskites under 290 nm excitation was also attributed to the weak absorption at high excitation energies.

The potential application of 0D perovskite quantum dots as emitting layer in color-converting devices has been explored in both colloidal and solid forms (thin films or powders); these systems are expected to have high converting efficiency similar to commercial color converter *viz.* phosphor.[^12^](#_ENREF_12)^,^[^13^](#_ENREF_13) A high PL QY has been achieved in perovskite colloids by employing ligands to stabilize samples of nanoscale size,[^14^](#_ENREF_14)^,^[^15^](#_ENREF_15) as well as thin films (solids) for microscale grain sizes.[^16^](#_ENREF_16)^,^[^17^](#_ENREF_17) Nevertheless, the QYs of colloids are severely quenched in their solid form, due to the aggregation originated from the loss of surface capping ligands.[^18^](#_ENREF_18) In addition, the abundance of grain boundaries is detrimental to the stability of the materials and in turn to the quantum yield of thin film perovskites.[^19^](#_ENREF_19) Hence, PL stability was achieved for perovskite solids by embedding them into stable inorganic and polymeric matrixes.[^12^](#_ENREF_12)^,^[^20^](#_ENREF_20) Nevertheless, the unique structure of 0D Cs_4_PbX_6_, with a wide band gap, spatial confinement induced by isolated lead halide octahedra, and high exciton binding energy, is expected to improve the stability of the materials and the PL QY of their solid form (powder/thin film), enabling their potential application in light-emitting devices.

The PL QY properties of undoped and Mn-doped Cs_4_PbX_6_ solids were investigated under 290 and 365 nm excitation (Supplementary Figure 11e and Supplementary Table 9). The QYs of all solids were significantly reduced compared to the corresponding colloidal form, due to the aggregation caused by loss of ligand and to the presence of surface trap states. Bright green emissions were observed for the Cs_4_PbBr_6_ solids, with a PL QY of 38% under 365 nm excitation. However, alike colloids, the total (host + Mn^2+^ emission) PL QY of 0D perovskite solids showed a six-fold increase upon doping in the mixed-halide (Br/Cl) solids, and an even higher fold increase in the chloride analogues. The Mn-doped Cs_4_Pb(Br/Cl)_6_ perovskite solids exhibited a maximum PL QY value of 9% under 365 nm excitation (Supplementary Figure 11). To the best of our knowledge, the PL QY of 21% is the highest reported to date for the Mn^2+^ orange emission in Cs_4_PbCl_6_ solids, due to enhanced energy transfer from host-related excitons to Mn^2+^ under 290 nm excitation and high exciton binding energy of the host. The large absorption by the wide band gap Cs_4_PbCl_6_ at high excitation energies enhances the PL QY of Mn^2+^ through efficient energy transfer from the host. An enhanced energy transfer from Cs_4_PbCl_6_ host to Mn^2+^ is the attribute of structural confinement of Cs_4_PbX_6_ perovskite arising from the isolated octahedra causing high exciton binding energy, in addition to the absorption strength of the host under the employed excitation wavelength.

**Supplementary Note 9.**

**Lifetime of Mn-doped Cs_4_Pb(Br/Cl)_6_ colloids.** Mn^2+^ doping in the perovskite nanocrystals has been reported to enhance the host emissions in the mixed halide (Br/Cl) and chloride analogues of perovskite. Moreover, the increment of host emission after Mn doping is more pronounced in the Br/Cl analogues than pure chloride one. The chloride doping is presumed to passivate the preexisting defects in the host responsible for the host PL efficiency. A similar trend in the PL results were observed in the D-state (host) emission of Cs_4_Pb(Br/Cl)_6_, which can be well explained by the life time of the D-state emission (Supplementary Figures 12 to 14). The chlorine passivation is further evidenced from the reduced contribution of the fast (non-radiative) component, associated with the decrease in the trap states. In addition, decline in the fast component associated to the trap state further confirms the passivation effect of chloride.

**Supplementary Note 10.**

Under high excitation energy (*λ*_ex_ of 290 nm), an enhanced Mn^2+^ PL QY of 27% was obtained for Cs_4_PbCl_6_:10% Mn, due to the efficient energy transfer from Pb^2+^ state to Mn^2+^ (Supplementary Figure 11d). This result is further supported by the decrease in the lifetime of Pb^2+^ emission (0.91 and 21.27 ns) for the doped Cs_4_PbCl_6_ relative to the undoped one (Supplementary Figure 15). The presence of two fast components in the Pb^2+^ emission at 375 nm under 300 nm excitation (the D-state is not excited) is consistent with the reported values for Pb^2+^ emission[^21^](#_ENREF_21).

**Supplementary References**

1. Palazon F*, et al.* Changing the Dimensionality of Cesium Lead Bromide Nanocrystals by Reversible Postsynthesis Transformations with Amines. *Chem Mater* **29**, 4167-4171 (2017).

2. Liu W*, et al.* Mn^2+^-Doped Lead Halide Perovskite Nanocrystals with Dual-Color Emission Controlled by Halide Content. *J Am Chem Soc* **138**, 14954-14961 (2016).

3. Parobek D*, et al.* Exciton-to-Dopant Energy Transfer in Mn-Doped Cesium Lead Halide Perovskite Nanocrystals. *Nano Lett* **16**, 7376-7380 (2016).

4. Mir WJ, Jagadeeswararao M, Das S, Nag A. Colloidal Mn-Doped Cesium Lead Halide Perovskite Nanoplatelets. *ACS Energy Lett* **2**, 537-543 (2017).

5. Liu H*, et al.* CsPb*_x_*Mn_1–_*_x_*Cl_3_ Perovskite Quantum Dots with High Mn Substitution Ratio. *ACS Nano* **11**, 2239-2247 (2017).

6. Li F*, et al.* High Br–Content CsPb(Cl_y_Br_1–y_)_3_ Perovskite Nanocrystals with Strong Mn^2+^ Emission through Diverse Cation/Anion Exchange Engineering. *ACS Appl Mater Interfaces* **10**, 11739-11746 (2018).

7. Zhang X*, et al.* Hybrid Perovskite Light-Emitting Diodes Based on Perovskite Nanocrystals with Organic–Inorganic Mixed Cations. *Adv Mater* **29**, 1606405 (2017).

8. Lindblad R*, et al.* Electronic Structure of CH_3_NH_3_PbX_3_ Perovskites: Dependence on the Halide Moiety. *J Phys Chem C* **119**, 1818-1825 (2015).

9. Akkerman QA*, et al.* Nearly Monodisperse Insulator Cs_4_PbX_6_ (X= Cl, Br, I) Nanocrystals, Their Mixed Halide Compositions, and Their Transformation into CsPbX_3_ Nanocrystals. *Nano Lett* **17**, 1924-1930 (2017).

10. Yin J*, et al.* Intrinsic Lead Ion Emissions in Zero-Dimensional Cs_4_PbBr_6_ Nanocrystals. *ACS Energy Lett* **2**, 2805-2811 (2017).

11. Guria AK, Dutta SK, Adhikari SD, Pradhan N. Doping Mn^2+^ in Lead Halide Perovskite Nanocrystals: Successes and Challenges. *ACS Energy Lett* **2**, 1014-1021 (2017).

12. Chen D, Wan Z, Chen X, Yuan Y, Zhong J. Large-scale room-temperature synthesis and optical properties of perovskite-related Cs_4_PbBr_6_ fluorophores. *J Mater Chem C* **4**, 10646-10653 (2016).

13. Kim YH*, et al.* A zero-thermal-quenching phosphor. *Nat Mater* **16**, 543 (2017).

14. Protesescu L*, et al.* Nanocrystals of Cesium Lead Halide Perovskites (CsPb*X*_3_, *X* = Cl, Br, and I): Novel Optoelectronic Materials Showing Bright Emission with Wide Color Gamut. *Nano Lett* **15**, 3692-3696 (2015).

15. Dirin DN*, et al.* Harnessing Defect-Tolerance at the Nanoscale: Highly Luminescent Lead Halide Perovskite Nanocrystals in Mesoporous Silica Matrixes. *Nano Lett* **16**, 5866-5874 (2016).

16. Cho H*, et al.* Overcoming the electroluminescence efficiency limitations of perovskite light-emitting diodes. *Science* **350**, 1222-1225 (2015).

17. Deschler F*, et al.* High Photoluminescence Efficiency and Optically Pumped Lasing in Solution-Processed Mixed Halide Perovskite Semiconductors. *J Phys Chem Lett* **5**, 1421-1426 (2014).

18. Saidaminov MI*, et al.* Pure Cs_4_PbBr_6_: Highly Luminescent Zero-Dimensional Perovskite Solids. *ACS Energy Lett* **1**, 840-845 (2016).

19. Berhe TA*, et al.* Organometal halide perovskite solar cells: degradation and stability. *Energy Environ Sci* **9**, 323-356 (2016).

20. Chen D, Fang G, Chen X. Silica-Coated Mn-Doped CsPb(Cl/Br)_3_ Inorganic Perovskite Quantum Dots: Exciton-to-Mn Energy Transfer and Blue-Excitable Solid-State Lighting. *ACS Appl Mater Interfaces* **9**, 40477-40487 (2017).

21. Zorenko Y, Gorbenko V, Voznyak T, Zorenko T. Luminescence of Pb^2+^ ions in YAG:Pb single-crystalline films. *physica status solidi (b)* **245**, 1618-1622 (2008).
